# Ocular Nanomedicine

**DOI:** 10.1002/advs.202003699

**Published:** 2022-02-12

**Authors:** Zhimin Tang, Xianqun Fan, Yu Chen, Ping Gu

**Affiliations:** ^1^ Department of Ophthalmology Shanghai Ninth People's Hospital Shanghai Jiao Tong University School of Medicine Shanghai Key Laboratory of Orbital Diseases and Ocular Oncology Shanghai 200011 P. R. China; ^2^ Materdicine Lab School of Life Sciences Shanghai University Shanghai 200444 P. R. China

**Keywords:** diagnostics, nanomedicine, ocular, ophthalmology, therapeutics

## Abstract

Intrinsic shortcomings associated with conventional therapeutic strategies often compromise treatment efficacy in clinical ophthalmology, prompting the rapid development of versatile alternatives for satisfactory diagnostics and therapeutics. Given advances in material science, nanochemistry, and nanobiotechnology, a broad spectrum of functional nanosystems has been explored to satisfy the extensive requirements of ophthalmologic applications. In the present review, the recent progress in nanosystems, both conventional and emerging nanomaterials in ophthalmology from state‐of‐the‐art studies, are comprehensively examined and the role of their fundamental physicochemical properties in bioavailability, tissue penetration, biodistribution, and elimination after interacting with the ophthalmologic microenvironment emphasized. Furthermore, along with the development of surface engineering of nanomaterials, emerging theranostic methodologies are promoted as potential alternatives for multipurpose ocular applications, such as emerging biomimetic ophthalmology (e.g., smart electrochemical eye), thus provoking a holistic review of “ocular nanomedicine.” By affording insight into challenges encountered by ocular nanomedicine and further highlighting the direction of future studies, this review provides an incentive for enriching ocular nanomedicine‐based fundamental research and future clinical translation.

## Graphical abstract

The distinctive functional ocular nanomedicine as potential alternatives for satisfying the requirements of abundant ophthalmologic applications has been comprehensively overviewed. In this comprehensive review, the authors summarize, discuss and highlight the fundamental principles on the design, fabrication, and ophthalmologic application of distinctive ocular nanomedicine, and provide an overview and deep discussion on the nano‐enabled amalgamation of ophthalmology, material chemistry, biology, and medicine.

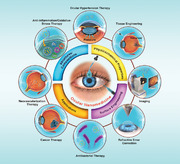

## 1 Introduction

The human eye is an exclusive and intricate organ of the visual senses.^[^ ^1^ ^]^ Anatomically, the human eye includes unique physiological barriers, including the tear film, cornea, and conjunctival barriers constituting the anterior segment barriers, and the inner limiting membrane and blood‐retinal barrier (BRB), which form the posterior segment barriers.^[^ ^2^ ^]^ Clinically, millions of individuals worldwide suffer from ocular diseases, including cataracts, dry eye, conjunctivitis, keratitis, myopia, glaucoma, ocular tumors, and vitreous/retinal diseases, especially age‐related macular degeneration (AMD).^[^ ^3^ ^]^ Most of these conditions can result in severe visual impairment and even blindness, thus substantially impacting the quality of life.^[^ ^3^ ^]^ The last few decades have witnessed a surge in pathogenic investigations to elucidate various pharmacological interventions for diverse ocular diseases. For example, dexamethasone has been widely employed as a highly effective anti‐inflammatory and antibacterial biopharmaceutical that inhibits ocular inflammatory and bacterial infections.^[^ ^4^ ^]^ However, owing to existing ocular barriers, intrinsic deficiencies of drug delivery by eye drops or intravitreal administration, such as poor permeation, ineffective distribution, and insufficient bioavailability, limit their clinical efficacy.^[^ ^5^ ^]^ Additionally, recombinant proteins/peptides, especially anti‐vascular endothelial growth factor (VEGF) agents (e.g., ranibizumab and aflibercept) with high potency and activity, minimize drug–drug interactions, and are routinely recommended to inhibit neovascular fundus diseases.^[^ ^6^ ^]^ However, these agents require intravitreal administration owing to their strong hydrophilicity and high molecular weight, which impedes their penetration across complex tissue barriers and cell membranes. Furthermore, their rapid physical and chemical degradation raises significant challenges in long‐term therapeutic effectiveness, warranting repeated intravitreal intervention that can induce intraocular bleeding, potential infection, and discomfort, leading to poor patient compliance.^[^ ^7^ ^]^ Importantly, the inability of available therapeutic strategies to image and diagnose ocular diseases early and precisely monitor post‐administration could result in unsatisfactory vision recovery.^[^ ^8^ ^]^ Therefore, it is urgent to explore safer and more efficient alternatives to combat eye‐related diseases.

With the rapid advancement of material science, nanochemistry, and nanobiotechnology, growing efforts have focused on developing safer and more effective ocular nanomedicines based on versatile nanosystems. Several nanomaterials, which are remarkably distinctive from their bulky counterparts, have been assessed to substantially modulate bioavailability, medical diagnostics, and ocular disease treatment. To a large extent, nanomedicine in ophthalmology can be attributed to its intrinsic therapeutic properties or drug/gene/cell delivery capability. Ocular nanomedicine can be elaborately engineered based on concrete biological scenarios (physicochemical cues, location, immunologic environment) to promote biostability and bioavailability in pathological regions. For instance, metal nanoparticles such as silver nanoparticles with intrinsic anti‐inflammatory, antibacterial, and antiangiogenic functions reportedly improve therapeutic efficacy in related ocular diseases.^[^ ^9^ ^]^ In terms of nanostructured systems, from traditional liposomes and polymers, emerging quantum dots (QDs) have been extensively used as ocular delivery carriers by expanding the encapsulated space and improving the bioavailability of ocular targeted drugs/molecules.^[^ ^10^ ^]^ Another considerable advantage of ocular nanomedicine is its ability to permeate across complex ocular barriers, especially the corneal‐retinal barrier and BRB, with minimal unwanted systematic/ocular side effects.^[^ ^4^ ^]^ Additionally, some engineered “smart” biomaterials, such as mesoporous silica nanoparticles (MSNs) with unique electronic, optical, and catalytic physiochemical properties, could trigger controllable drug release by responding to exogenous physical irradiation (e.g., light and ultrasound) or endogenous biological stimulations (e.g., redox and pH).^[^ ^11^ ^]^ Moreover, numerous nanomaterials utilized for sensing, imaging, and labeling biomarkers or cells/tissues involved in eye‐related diseases were deemed both evolutionary and revolutionary, along with significant success achieved in nanotechnology.^[^ ^12^ ^]^ For instance, nanomaterials, particularly inorganic nanoparticles, ranging from 1 to 100 nm, are comparable in size to peptide drugs and various cellular compartments (e.g., mitochondria) and exhibit a large surface area and high intracellular biodistribution, which is beneficial for in vivo imaging, biosensing, and non‐invasive tracking.^[^ ^13^ ^]^ In ophthalmology, routine use of ocular fluorophores under visible light irradiation may lead to ocular autofluorescence, thereby decreasing the image contrast. Multifunctional nanomaterials such as upconversion nanoparticles and QDs allow fluorescence imaging under the emission of visible and near‐infrared (NIR) light without causing ocular autofluorescence.^[^ ^1^, ^14^ ^]^

**Figure 1:**
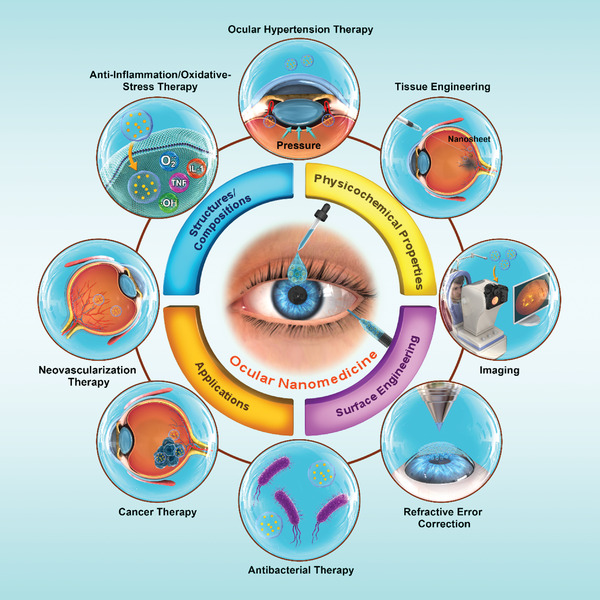
Schematic illustration of ocular nanomedicine for versatile biomedical applications in opthalmology.

Herein, we attempted to provide a holistic overview of nanomedicine used in ophthalmology by examining state‐of‐the‐art literature mainly reported between 2016 and 2021. Accordingly, we comprehensively reviewed the recent progress in ocular nanomedicines composed of conventional biomaterials (e.g., liposomes, polymers, metal, and metal oxide nanoparticles) and emerging nanomaterials (e.g., QDs and exosome‐based nanomaterials). Furthermore, efforts have been made to demonstrate their fundamental physicochemical properties, especially size, surface charge, hydrophilicity, hydrophobicity, and biodegradability, which are strongly associated with therapeutic effectiveness in ocular biological/pathological milieu. In addition, developmental strategies for the surface engineering of ocular nanomedicine have been discussed in detail, facilitating personalized ocular medicine, including controllable and targeted release, on‐demand gene delivery, pathology‐oriented diagnostics and therapeutics (theranostic), and side‐effect mitigation for specific paradigms. Furthermore, we emphasized their versatile ophthalmologic applications, as illustrated in **Figure** **1**, especially demonstrating biomimetic ophthalmology, for example, smart electrochemical eye. Finally, inspired by current trends and therapeutic concepts, we highlighted challenges encountered during ocular nanomedicine and corresponding directions, such as the development of non‐invasive intraocular penetrable nanomaterials or nanomaterials with retrieval and recycling capability (e.g., ocular microrobots). Although the role of ocular nanomedicine remains only partially elucidated, it is highly expected that ocular nanomedicine will confer marked contributions to ocular disease management based on advanced concepts and improved theranostic performance.

## 2 Structures/Compositions of Ocular Nanomedicine

The family of nanomaterials has enriched diverse nanomedical applications, given their diverse structures and compositions. The most relevant formulations in ocular nanomedicine are composed of synthetic organic nanomaterials (including lipid‐based nanoparticles, dendrimers, polymeric micelles, polyester, and natural biopolymer), inorganic nanomaterials (e.g., metal [oxide] nanoparticles, QDs, carbonous and silica nanoparticles [SiNPs]), and biological components of purified biomolecules (e.g., exosomes). The present section summarizes various ocular nanomedicines characterized by distinct compositions, unique structures, and properties based on recent representative studies, discussing their respective merits/demerits when utilized in the ophthalmologic field.

### 2.1 Lipid‐Based Nanoparticles

**Figure 2:**
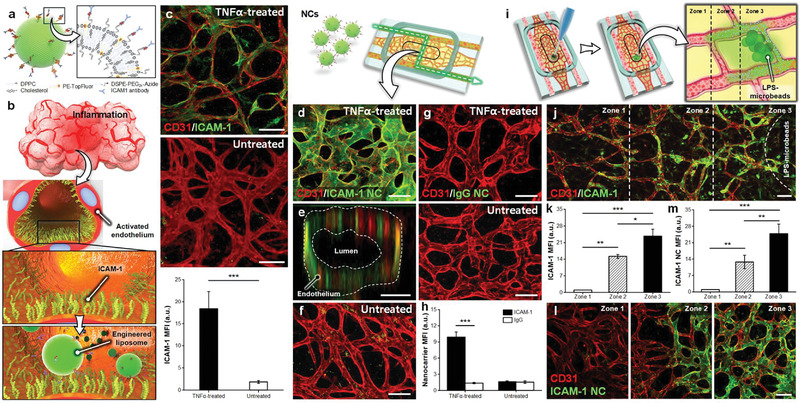
The active targeting capability of liposomal nanoparticles. a,b) A schematic illustration of functionalized fluorescent dipalmitoylphosphatidylcholine‐cholesterol liposomes by coupling with monoclonal antibody against intercellular adhesion molecule 1 (ICAM‐1) to target the ICAM‐1 on the activated endothelial surface. c) Intravascular flow of tumor necrosis factor (TNF) for 6 h caused endothelial expression of ICAM‐1. Scale bar, 100 µm. d,e) Anti‐ICAM‐1 nanocarriers binded to the TNF‐treated endothelial surface. Scale bars, 100 µm in (d) and 25 µm in (e). f) Non‐TNF‐activated blood vessels presented minimal liposome integration. Scale bar, 100 µm. g,h) IgG‐coupled liposomes did not adhere to the blood vessels. Scale bar, 100 µm. i) Local inflammation was induced by directly injecting lipopolysaccharide‐soaked microbeads into vascularized hydrogel via opening of the culture chamber. j) The vascularized hydrogel after incubation of lipopolysaccharide (LPS)‐laden microbeads clearly showed the spatially graded activation of ICAM‐1. k) The expression level of ICAM‐1 was the highest in the vicinity of LPS‐microbeads and decreased gradually with increasing distance from the source of inflammation. The expressed level was inversely proportional to the distance from the lipopolysaccharide microbeads. Scale bar, 100 µm. l,m) Adhering to anti‐ICAM‐1 nanoparticles perfused by vasculature corresponds to the spatial pattern of ICAM‐1 stimulation, further revealing the active targeting ability of functionalized liposomal nanoparticles. Scale bar, 100 µm. Adapted with permission.^[^ ^18a^ ^]^ Copyright 2019, American Chemical Society.

Lipid‐based nanoparticles can be employed as colloidal systems and formulated using typical liposomes, solid lipid nanoparticles (SLNs), and nanostructured lipid carriers (NLCs). Liposomes, as non‐covalent aggregates, are composed of one or more phospholipid bilayer membranes and are thus capable of encapsulating both hydrophilic and/or lipophilic biopharmaceuticals.^[^ ^15^ ^]^ Liposomes have been widely developed for treating the anterior eye segment by topical instillation. Accordingly, cationic liposomes form ionic interactions with the cornea, resulting in high corneal drug absorption by prolonging drug residence time, along with low toxicity and antigenicity.^[^ ^16^ ^]^ However, drug delivery into the posterior eye segment, especially the retina, remains challenging due to complex ocular barriers. To improve cellular localization retinal distribution, light‐activated liposomes have been formulated using time‐ and site‐specific drug delivery into intraocular tissues.^[^ ^10^, ^17^ ^]^ Moreover, liposomal nanoparticles could afford focused protection against ocular diseases by targeting specific signaling (e.g., disease driver common to different types of retinal diseases) or by engineering with a specific antibody to target the pathophysiological niche of unhealthy tissues.^[^ ^18^ ^]^ Accordingly, Paek et al. first designed azide‐functionalized fluorescent dipalmitoylphosphatidylcholine‐cholesterol liposomal nanoparticles. The liposomal surface was subsequently functionalized with a monoclonal antibody against intercellular adhesion molecule 1 (ICAM‐1) to confer active targeting abilities for specifically binding to induced endothelial cells in the inflamed vasculature (**Figure** **2**).^[^ ^18a^ ^]^ Interestingly, biocompatible liposomes for different drug delivery have been verified in several clinical trials to combat several ocular diseases, including glaucoma, retinoblastoma, and metastatic malignant uveal melanoma (Table 2).^[^ ^19^ ^]^ However, it should be noted that potential shortcomings of liposomes, such as the formation of lipid crystal matrix, gelation tendency, in vivo burst release of cargo, and oxidation of liposomal phospholipids, may result in poor shelf‐life stability, insufficient drug loading, poor batch‐to‐batch reproducibility, and high‐cost manufacturing of liposomal products, which should be overcome in the future.^[^ ^20^ ^]^

Lipid nanoparticles, especially SLNs and NLCs, are important alternatives to liposomes, exhibiting an overwhelming advantage over liposomes in terms of cost‐effective manufacturing, easy scale‐up, and fewer drug‐leakage issues. Compared with the liquid state, SLNs are composed of physiological and biocompatible solid lipids such as triglycerides, steroids, and fatty acids, thereby providing high superiority to avoid organic solvent formulation.^[^ ^21^ ^]^ However, a short‐term release window (3 h to 2 weeks) and inadequate therapeutic concentrations of substrates in ocular tissues, such as the interface membrane of tear film/cornea, vitreous body, and retina, have been reported after SLN delivery by parenteral and topical administration.^[^ ^22^ ^]^ Moreover, some additional drawbacks of SLNs, including limited biopharmaceutical loading and rapid elimination/metabolism by the mononuclear phagocytic system, need to be overcome. As second‐generation lipid nanocarriers, NLCs are composed of biocompatible surfactants, drugs, lipids, or solid lipids. In contrast to SLNs, NLCs are preferred to afford pharmaceutical protection and entrapment efficiency by overcoming expulsion during phase modifications, low drug loading, and high water content of aqueous dispersions.^[^ ^23^ ^]^ To date, both NLCs and SLNs have shown potential for small molecule delivery into various ocular tissues; however, the capacity of SLNs to deliver peptide‐ and protein‐based biomacromolecules into ocular tissues needs to be further explored.

### 2.2 Polymeric Micelles

**Figure 3:**
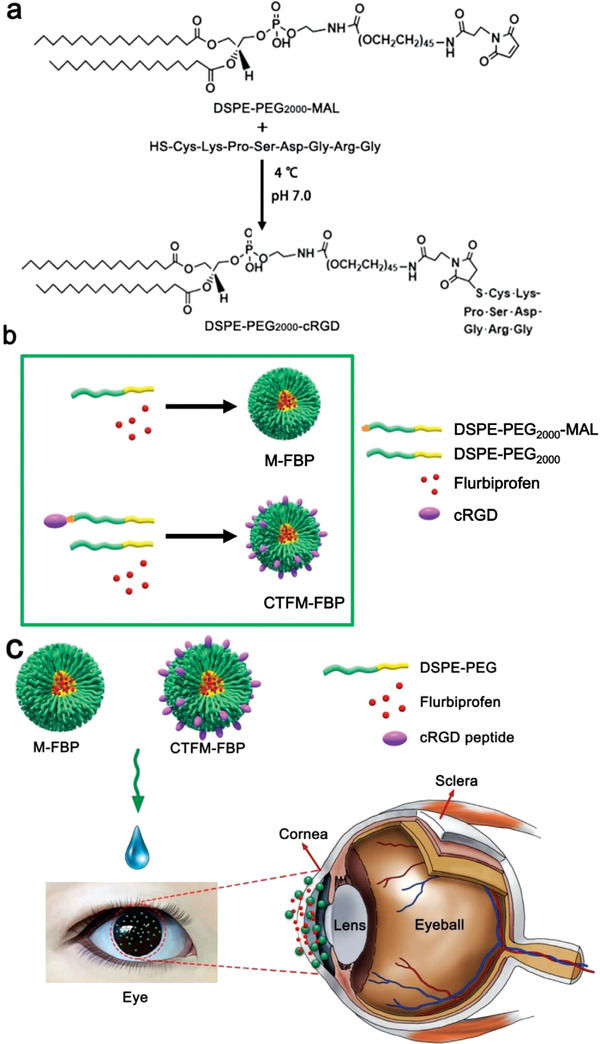
Formulation and application of flurbiprofen sodium (FBP)‐encapsulated nanomicelles. a) 1, 2‐distearoyl‐sn‐glycero‐3‐phosphoethano–lamine‐*N*‐[maleimide (polyethylene glycol)‐2000] (DSPE‐PEG_2000_‐MAL) and cyclic peptide ligand c (cRGD) peptide to fabricate DSPE‐PEG_2000_‐cRGD. b) Formulation of FBP‐loaded nanomicelles (M‐FBP) and cornea‐targeting peptide‐functionalized nanomicelles (CTFM‐FBP). c) Schematic illustration of nanomicelle‐facilitated targeted ocular delivery. Reproduced under the terms of the Creative Commons CC‐BY license.^[^ ^28^ ^]^ Copyright 2018, The Authors. Published by Wiley‐VCH.

Polymeric micelles (10–100 nm) comprise block copolymers with amphiphilic components, typically enabling self‐assembly under aqueous conditions to generate organized supramolecular/core–shell structures.^[^ ^24^ ^]^ Over the past several years, polymeric micelles, especially those formulated using poly(lactide), acrylic acid, and vinylpyrrolidone, have been routinely used as nanocarriers to improve therapeutic outcomes in different ocular diseases.^[^ ^25^ ^]^ Given their ability to stabilize and solubilize hydrophobic compounds, polymeric micelles reportedly enhance drug permeation by prolonging retention on the ocular surface.^[^ ^26^ ^]^ Facile surface modification and targeted delivery to improve drug bioavailability confer additional advantages to polymeric nanoparticles used in ophthalmology.^[^ ^27^ ^]^ For instance, 1, 2‐distearoyl‐sn‐glycero‐3‐phosphoethano–lamine‐*N*‐[maleimide (polyethylene glycol)‐2000] (DSPE‐PEG_2000_‐MAL) was functionalized using a cyclic peptide ligand (GRGDSPKC) (cRGD) to develop a tailored DSPE‐PEG_2000_‐cRGD nanomicelle for encapsulating flurbiprofen. Combining cRGD with integrin receptors on the corneal surface, the fabricated nanomicelle could specifically target the cornea, facilitating robust and rapid mucoadhesion, superior transcorneal penetration performance, and ocular surface retention of nanomicelles (**Figure** **3**).^[^ ^28^ ^]^ Given the multiplicity of functional groups, some proposed micelle nanocarriers exhibit various polymer block arrangements based on diverse requirements and can deliver poorly aqueous soluble drugs by solubilizing the hydrophobic core. Additionally, this overwhelming advantage provides some important implications for the further development of ophthalmic nanoformulations. Interestingly, small‐molecule drugs such as cyclosporine A have been developed into nanomicelles and extensively studied in clinical trials for efficient control of dry eyes, further suggesting the enormous market for nanomicellar‐based products in ocular nanomedicine (Table 2).^[^ ^29^ ^]^ However, considerable efforts should be made to overcome the limitations of self‐assembled polymeric micelles prior to extensive application in ophthalmology, such as lack of potential and adequate utilization in gene delivery, limited entrapment of macromolecule agents, and unsuitable scale‐up methods.

### 2.3 Dendrimers

**Figure 4:**
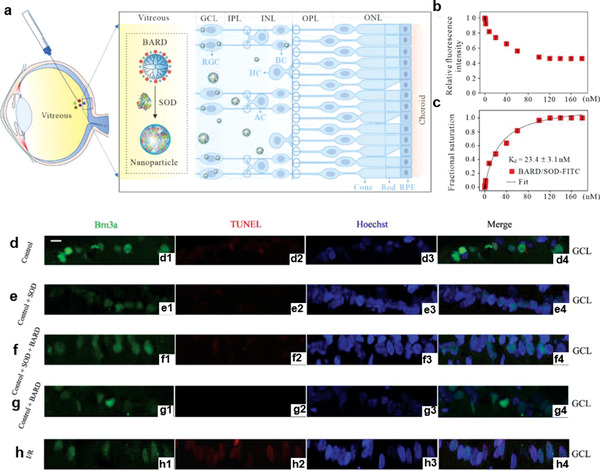
Preparation and application of boronic acid‐rich dendrimer (BARD)‐mediated intracellular superoxide dismutase (SOD) delivery. a–c) Intravitreal injection of BARD/SOD nanoformulations for intracellular SOD delivery. a) The eyeball structure from outside to inside were enlarged, and presented from right to left. b) Binding curve and c) fractional saturation curve of BARD/SOD‐FITC nanoformulations at different polymer concentrations, and SOD‐FITC was fixed at 176 nm. *n* = 3, data presented as mean ± SD. d–h) Confocal microscope images of Brn3a (green), TUNEL (red), Hoechst (blue), and merged staining in the ganglion cell layer (GCL). d) Normal control rat; e) normal rat injected with SOD; f) normal rat injected with BARD/SOD nanoformulation; g) normal rat injected with BARD; h) ischemia/reperfusion rat. Scale bar: 20 µm. RGC, retinal ganglion cell; BC, bipolar cell; HC, horizontal cell; AC, amacrine cell. ONL, outer nuclear layer; OPL, outer plexiform layer; INL, inner nuclear layer; IPL, inner plexiform layer. Reproduced with permission.^[^ ^33^ ^]^ Copyright 2020, Elsevier Ltd.

Unlike linear polymers, dendrimers with branched and layered tree‐like topologies can be divided into several generations depending on the number or size of branches and functional groups at the terminal surface.^[^ ^30^ ^]^ These versatile dendrimer topologies confer excellent control over dispersity, stability, specific size, molecular weight, distribution, solubility, and biological activity during dendrimer synthesis. Interestingly, dendrimers with multivalent characteristics endow higher payloads of small biological molecules, biocompatible pharmaceuticals, and imaging agents.^[^ ^31^ ^]^ Currently, poly(amidoamine) (PAMAM)‐derived dendrimers are the most commonly applied and commercially available systems for ocular applications, conferring enhanced drug bioavailability, tolerability, and biological response and diminishing drug clearance from the body following subconjunctival injection.^[^ ^32^ ^]^ Recently, Zhou et al. rationally constructed a boronic acid‐rich dendrimer (BARD) based on generation 5 PAMAM dendrimers for ocular intracellular superoxide dismutase (SOD) delivery for oxidative stress reduction in an acute retinal ischemia/reperfusion injury rat model. The authors revealed that the BARD‐mediated SOD nanoformulation could efficiently protect retinal function and reduce cell apoptosis by achieving high levels of cellular uptake without immunogenicity and cytotoxicity, thus suggesting that the PAMAM dendrimer‐based nanoformulation possesses robust efficacy for the intracellular delivery of native peptides and proteins without impairing bioactivities (**Figure** **4**).^[^ ^33^ ^]^ In addition, it is considerably flexible for precisely regulating the delivery of functional dendrimers of diverse biomedical substances by interacting with highly functional amino, carboxyl, and/or hydroxyl groups on the terminal surface of dendrimers. Using co‐modification with a penetrating and cyclic RGD hexapeptide, a dendrimer‐based PAMAM system has been developed for non‐invasive, targeted penetration into the posterior ocular segment.^[^ ^34^ ^]^ Dendrimer‐based vehicles also exhibit powerful intrinsic anti‐inflammatory and antioxidant activities in inflamed regions, which could effectively deliver therapeutic substrates to the inflamed ocular tissue for improved therapeutic outcomes.^[^ ^35^ ^]^ Nonetheless, several underlying disadvantages of dendrimers in terms of toxicological issues, complex multistep synthesis, inadequate evaluations of in vivo quality control, and high cost of preparation significantly prohibit the progression and advancement of dendrimers from laboratory to clinic settings.

### 2.4 Polyester Nanoparticles

**Figure 5:**
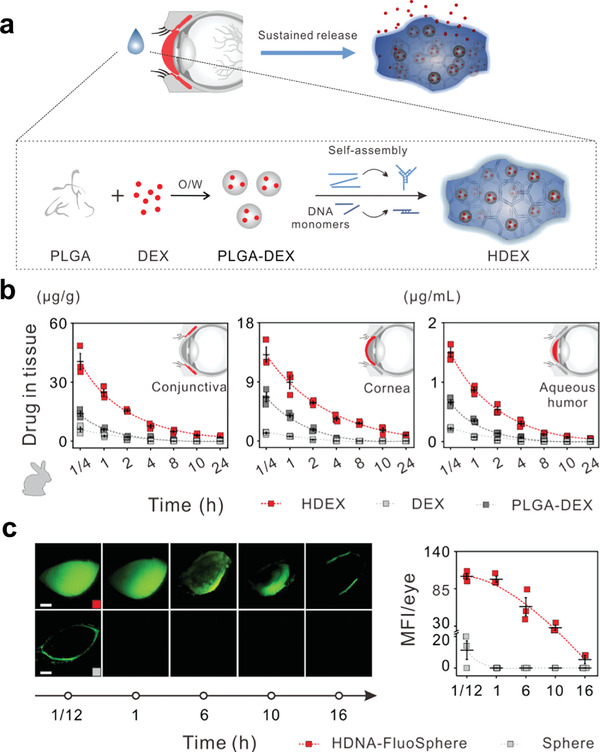
Preparation and application of DNA/poly(lactic‐*co*‐glycolic acid) (PLGA) hybrid hydrogel (HDNA). a) Schematic illustration showed the complexation of dexamethasone (DEX) with HDNA (HDEX)‐ and HDNA‐mediated sustained DEX release. b) Rabbits were topically treated with indicated materials and then were collected at 1/4, 1, 2, 4, 8, 10, and 24 h after treatment to detect the drug in tissue. c) Rabbits were topically treated with HDNA‐FluoSpheres or Fluo‐Spheres. FluoSpheres were entrapped in DNA hydrogel or PBS instead of PLGA nanoparticles. Representative fundus camera images of rabbits at 1/12, 1, 6, 10, and 16 h after instillation. Scale bars: 0.5 cm. Reproduced with permission.^[^ ^4^ ^]^ Copyright 2019, American Chemical Society.

Polyester nanoparticles are comparatively stable in vivo, tunable, and reproducible and have been positioned to provide useful solutions to a wide range of medical challenges. In ophthalmology, extensively available polyesters such as poly(lactide‐*co*‐glycoside) (PLGA), polylactic acid (PLA), and poly(*ε*‐caprolactone), exhibiting highly favorable biocompatibility and biodegradability, have been extensively employed to synthesize nanoparticles, tissue‐engineered implants, and surgical sutures.^[^ ^36^ ^]^ In particular, PLGA nanoparticles, which are considered the least toxic, biodegradable synthetic polymers by simple elimination, have been widely employed in ophthalmology for high drug loading, efficient delivery, and long‐term release of diverse cargo molecules, ranging from hydrophobic/hydrophilic small molecules to large biopharmaceuticals.^[^ ^36^, ^37^ ^]^ For example, a distinctive DNA/PLGA hybrid hydrogel (HDNA) with a porous structure effectively loaded water‐insoluble dexamethasone for sustained delivery for at least 4 weeks to attenuate ocular inflammatory symptoms following retinal injection (**Figure** **5**).^[^ ^4^ ^]^ In addition to the various properties discussed, polyester nanoparticles can target delivery into intended intracellular sites/tissues and release the loaded cargo in a sustained fashion. A brief, low‐power, far‐ultraviolet light‐responsive degradable polymer PLGA allowed on‐demand delivery of anti‐angiogenesis molecules, thus suppressing choroidal neovascularization (CNV) in rats. This nanosystem stably retained the encapsulated molecules in the vitreous for up to 30 weeks post‐injection, thereby non‐invasively controlling the timing of drug release in response to 365 nm ultraviolet exposure.^[^ ^38^ ^]^ Collectively, synthetic polyester polymers exhibit a precisely controlled chemical structure and biological inertness, thus possessing great chemical versatility and high batch‐to‐batch uniformity. Nonetheless, polyester‐based nanosystems lack biological cues, given their biologically inert characteristics, which limits further progress. Accordingly, incorporating functionalities into the polyester structure to improve interactions with targeted cells/tissues remains an essential topic in the ocular and polymeric science field.

### 2.5 Natural Polymers

Naturally occurring polymers are characterized by cell‐activated proteolytic degradation, bioactivity, very low/even no toxicity profiles, and enhanced membrane permeability.^[^ ^39^ ^]^ Natural biopolymer‐based nanomedicines used for ocular diseases are mainly derived from gelatin, chitosan, and its derivatives, for example, hyaluronic acid (HA). Among them, gelatin‐based hydrogels perfectly mimic the natural dermal extracellular matrix and are often used to support ocular tissue regeneration.^[^ ^40^ ^]^ Different technologies can readily modify mechanical, biodegradable, and biological properties in the developed cross‐linkable gelatin. For instance, an injectable, photo‐curable gelatin system was fabricated using thiolated gelatin and acrylated gelatin with tunable mechanical properties. The developed system can be employed as a potential cell‐supportive scaffold for focal corneal wound repair, and ultraviolet irradiation showed no obvious harmful effects on ocular tissues. The mechanical properties of generated hydrogels could be readily modified (0.3–22 kPa of the post‐cure shear modulus) by varying the photo intensity, the ratio of acrylate to thiol groups, and solid content. Moreover, the biodegradation times could be tuned by altering the solid content.^[^ ^41^ ^]^ Additionally, a recent study has reported that an injectable positive‐charge‐tuned gelatin‐tyramine hydrogel with cross‐linking ability was highly effective for siRNA delivery and protection. The carriers significantly reduced subconjunctival scarring in a rabbit model after glaucoma filtration surgery without cytotoxicity. Typically, a novel delivery system based on a charge‐tunable gelatin hydrogel is highly scalable and simple to fabricate, exhibiting strong translational potential for epigenetic silencing therapy.^[^ ^42^ ^]^

**Figure 6:**
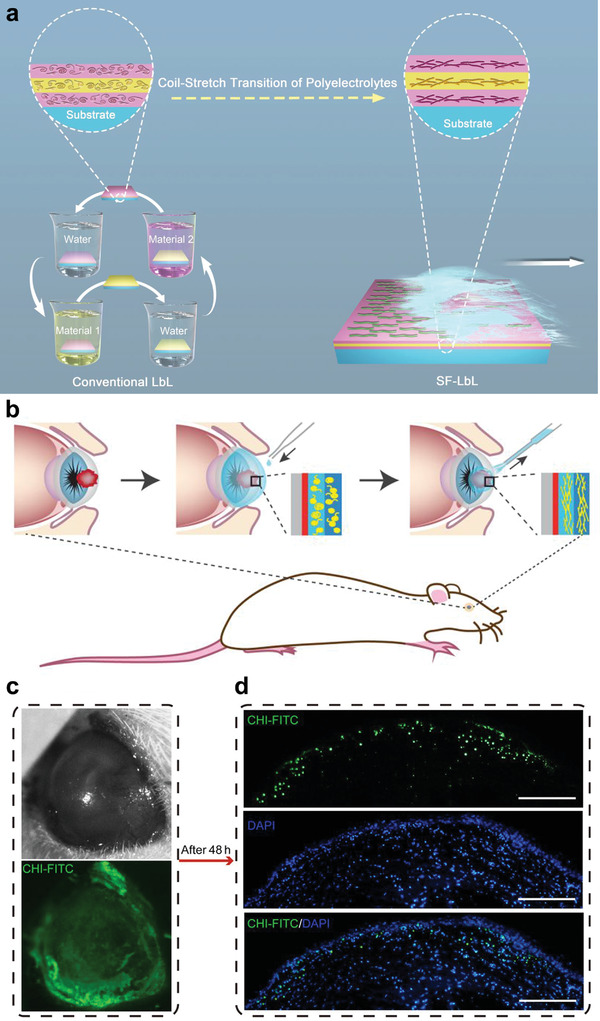
Chitosan (CHI)‐based self‐assembly nanofilms for corneal wound repair. a) Schematic illustration of conventional layer‐by‐layer (LbL) and shear flow‐driven LbL (SF‐LbL) assembly processes. Polymer molecules mainly showed a random coil structure in static solution, but achieved a more stretched structure in flow state via coil‐stretch transition. b–d) Remodeling of acellular matrix of the porcine cornea and modification of a rat eye in situ by SF‐LbL‐assembled coating strategy. b) Schematic illustration of (CHI‐FITC/hyaluronic acid (HA))_15_ film fabrication on a wounded porcine eye in situ. c) Bright‐field (top) and fluorescence (bottom) photos of an alkali‐burned rat eye covered with SF‐LbL‐assembled (CHI‐FITC/HA)_15_ films. d) Fluorescence images of a repaired rat cornea in histologic section. Scale bars = 200 µm. Reproduced with permission.^[^ ^45^ ^]^ Copyright 2019, American Chemical Society.

Chitosan possesses abundant functional groups that vary in composition, including hydroxyl, carboxyl, and amino groups that interact with mucosal surfaces to afford mucoadhesion by hydrogen bond formation.^[^ ^43^ ^]^ The mucoadhesive property allows nanoparticles to markedly prolong the retention time in ocular tissues and impede drug clearance.^[^ ^44^ ^]^ For example, DexaSite has shown success in clinical trials for treating postoperative inflammation and pain following ocular surgery (NCT03192137), which is mainly attributed to the addition of chitosan to achieve greater viscosity for effective dexamethasone delivery. To improve the time‐consuming and laborious nature of the polysaccharide fabrication process, functional drug‐free and shear flow‐driven layer‐by‐layer (SF‐LbL)‐assembled nanofilms consisting of chitosan and heparin were successfully constructed for corneal modification and defective wound healing. This strategy is expected to afford a versatile and robust nanoplatform for nanofilm surface engineering in ocular nanomedicine (**Figure** **6**).^[^ ^45^ ^]^ Depending on advantages such as low immunogenicity, high transfection efficacy, and lack of mutational possibility in contrast to virus vectors, chitosan has also been identified as a potential non‐viral nanoplatform for gene delivery.^[^ ^46^ ^]^

HA is a biodegradable biopolymer that naturally exists within the vitreous of the eye, rendering it non‐immunogenic and highly biocompatible when employed in biomedical systems. Importantly, HA also exhibits ligands for receptors, such as CD44, located in retinal cells, which is particularly significant for ocular delivery.^[^ ^10a^ ^]^ However, HA alone cannot form a gel; thus, hydrogels made from HA depend on cross‐linking or gelling agents and chemical modifications as both static and stimuli‐responsive. For instance, HA with several anionic carboxylic groups can be added to cationic chitosan to form chitosan‐based hybrid hydrogels (chitosan‐HA) by ionic interactions, effectively improving the drug loading efficiency and cargo delivery to the conjunctiva and cornea without apparent ocular irritation.^[^ ^47^ ^]^ HA hydrogels have been investigated as an ocular delivery system and an artificial vitreous substitute by chemical or enzymatic cross‐linking.^[^ ^48^ ^] ^ Baker et al. produced a vitreous substitute comprising a novel hyaluronan‐oxime cross‐linked hydrogel by chemical modification, exhibiting physical features similar to those of the native vitreous, including density, refractive index, and transparency. The hydrogel also showed no impairment in retinal function after implantation for over 90 days when compared with eyes administered balanced saline solution, thus significantly enhancing the status quo as a vitreous substitute.^[^ ^48a^ ^]^ Although various biodegradable natural polymers have been successfully utilized in several preclinical trials, few passively targeted nanocarriers have been applied for ocular clinical application. In addition, natural polymers such as ocular nanocarriers remain challenging in terms of chemical synthesis, precise purity, and identification of chemical structure/composition, thus inducing batch‐to‐batch variability.

### 2.6 Inorganic Nanomaterials

Although most organic nanosystems display high biocompatibility in ophthalmologic applications, their relatively low chemical and thermal stability may hinder further development. Conversely, a vast array of rapidly progressing inorganic nanoparticles, ranging from metal nanoparticles, metal oxides, semi‐conductive QDs, graphene oxide, and SiNPs, exhibit unique intrinsic properties, especially high physiological stability, tunable morphology and structure, and easy functionalization. These inorganic nanoparticles have attracted extensive attention from the scientific community for expanding ophthalmological applications, especially wearable digital vision systems.

**Figure 7:**
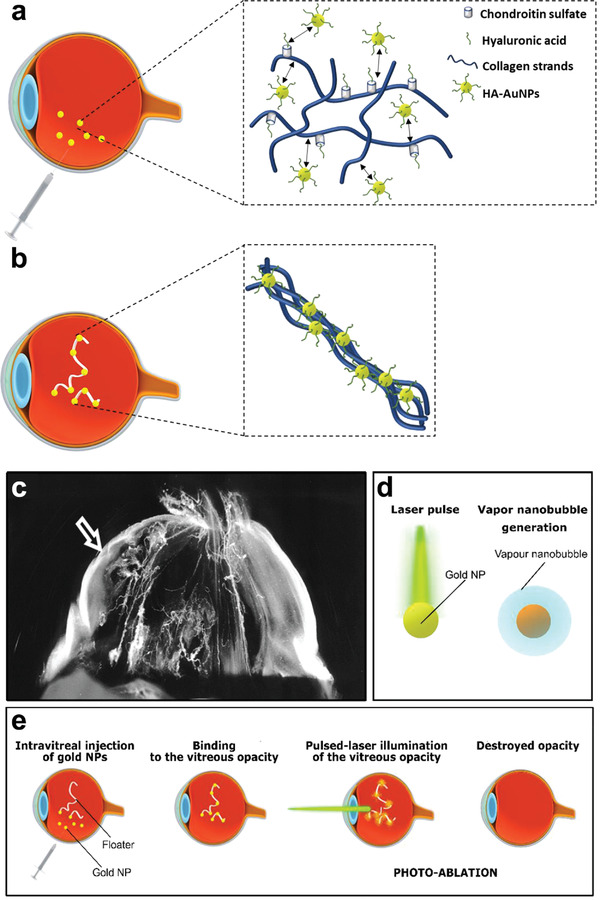
Photoablation by light‐triggered vapor gold nanoparticles (AuNPs)‐based nanobubbles for human vitreous opacity therapy. a) Schematic illustration of the fate of HA‐coated AuNPs in vitreous. b) Schematic illustration of binding of AuNPs and HA to a vitreous opacity. c) Dark‐field microscopy of vitreous in an 88‐year‐old patient showed that the vision‐disturbing floaters were caused by the age‐related vitreous collagen aggregation into visible fibers in central vitreous body. Floaters could also be caused by the dense collagen matrix in outer vitreous body (arrow) after posterior vitreous detachment. d) Schematic illustration of the production of vapor nanobubbles after AuNPs illumination by a pulsed laser. e) Schematic illustration of the concept of “AuNPs‐assisted photoablation”: AuNPs bind to the opacities by intravitreal injection, and then local pulsed‐laser illuminates to generate vapor nanobubbles, which mechanically ablate the vitreous opacities. Reproduced with permission.^[^ ^51^ ^]^ Copyright 2019, American Chemical Society.

Metal nanoparticles, especially gold nanoparticles (AuNPs) of different shapes, are widely accepted as indispensable high‐contrast agents for photothermal therapy, bioimaging, and biosensing to manage ocular disorders.^[^ ^49^ ^]^ Given the high efficacy of photothermal conversion using palladium‐coated gold nanorods (GNRs@Pd), GNRs@Pd and gelatin were selected as raw materials to develop a photothermal conversion gelatin hydrogel‐based mini‐eye patch. Following adherence to the lacrimal gland, the GNRs@Pd hydrogel eye patch could sense diverse visible light irradiations and spontaneously respond by heating up to secrete more tears, which is beneficial for preventing dry eye, as well as for avoiding preservatives contained in artificial tears.^[^ ^50^ ^]^ Additionally, based on the remarkable plasmon properties of AuNPs, Wagle et al. designed an AuNP support platform with HA coating to generate vapor nanobubbles on applying mechanical forces exerted by pulsed laser illumination. The results revealed that the nanobubbles could successfully destroy collagen aggregates with ≈1000 times less light energy and prevent damage to normal tissues, exhibiting easier, faster, and safer behavior than typical YAG laser therapy (**Figure** **7**).^[^ ^51^ ^]^ Like AuNPs, a series of reports have suggested that silver nanoparticles (AgNPs) can be extensively applied in ocular diseases. For instance, AgNPs could serve as ocular bandages to kill infectious pathogens in bacterial keratitis and promote cell proliferation for ocular wound healing.^[^ ^52^ ^]^ As an effective antioxidant, AgNPs were also efficacious in preventing experimental selenite‐induced opacification of lenses.^[^ ^53^ ^]^ It has also been reported that AgNPs exert potent antiangiogenic activity and anticancer effects by suppressing cell survival during ocular neovascularization and tumor treatment.^[^ ^54^ ^]^ These findings suggest that AgNPs could serve as a promising candidate for managing various ocular diseases. Furthermore, nanoceria and fabricated nanomaterials reportedly possess autoregenerative and catalytic features and have been widely used as non‐enzymatic antioxidants to alleviate ocular oxidative stress and inflammation, especially for treating ocular surface diseases and long‐term reduction of intraocular pressure (IOP).^[^ ^55^ ^]^ These ideal outcomes revealed that metal‐ and metal oxide‐based nanoparticles could act as effective therapeutic agents for managing ocular diseases; however, their potential toxic effects on the normal retina or whole eye need to be resolved.^[^ ^56^ ^]^

**Figure 8:**
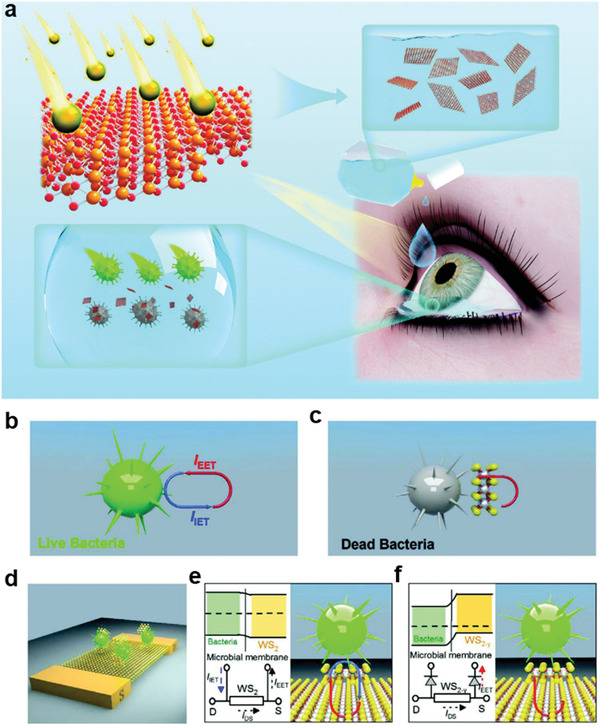
Vacancy‐induced antibacterial activity of two‐dimensional transition metal dichalcogenides (XS_2_) quantum dots (QDs) against drug‐resistant bacteria. a) Schematic diagram of vacancy‐induced antibacterial activity of WS_2‐y_ (remaining sulfur vacancies and reducing sulfur content) QDs for bacterial keratitis treatment. Antibacterial mechanism of WS_2–y_ QDs. b) Schematic illustration of cellular metabolism. Intracellular and extracellular electrons transfer (IET and EET) produce an electron loop between intracellular and extracellular environment, and the respiratory proteins on microbial membrane work as an electron conduit. c) Disturbed cellular metabolism by WS_2–y_ QDs. WS_2–y_ limited the electronic transfer, disturbed cellular metabolism, and triggered microbe death. d) Schematic diagram of the WS_2_ field‐effect‐transistor biosensor. e,f) Schematic circuitry to illustrate the proposed mechanism for different responses of bacteria to WS_2_ and WS_2–y_ QDs, respectively. In contrast to *Staphylococcus aureus* solution, the current is little changed in WS_2_ (S_0_) QDs and *S. aureus* solution, suggesting a still balance of IET and EET processes. However, a significant current decreasing was observed in WS_2–y_ (S_1_–S_5_), demonstrating the current for the IET process is blocked. Thus, the bactericidal activity of WS_2–y_ stemmed from the blocked ET on the microbial membrane. Reproduced with permission.^[^ ^57^ ^]^ Copyright 2020, Wiley‐VCH.

Emerging QDs allowing easy conjugation, cost‐effective production, and stable excitable fluorescence have gained momentum in ocular bioimaging and cell/tissue labeling, electrical stimulation, or targeted delivery.^[^ ^8^, ^10^, ^14^ ^]^ Owing to the rapid development of nanotechnology, 2D transition metal dichalcogenide (XS_2_, X = Mo/W) QDs have shown significant potential for combating drug‐resistant bacteria by ion irradiation. During this process, sulfur atoms in the top layer of XS_2_ were sputtered, leaving S‐vacancies and tuning the atomic ratio of S:X (XS_2–y_). S‐vacancies generated more surface electronic states, improving the quantity of charge transport on the surface of QDs; the physical contact of the microbe membrane with WS_2–y_ QDs (p‐type semiconductor) resulted in p‐n junctions, limiting the one‐way charge transport. Thus, WS_2–y_ QDs exhibited strong reactive oxygen species (ROS)‐independent oxidative stress, with a rapid response and independent of light activation (**Figure** **8**).^[^ ^57^ ^]^ Although 2D transition metal nanosheets are regarded as one of the most promising candidates in ophthalmology owing to their intriguing optical properties, large surface area, and easily functionalized surface, their biosafety and long‐term effectiveness in vivo need to be further explored. Recently, a flexible optoelectronic sensor array using a combination of indium‐based colloidal QDs or perovskite QDs (as active materials) was designed for an efficient neuromorphic vision system.^[^ ^58^ ^]^ The practical artificial device possesses an extraordinary sensitivity to light and a specific detectivity, demonstrating neuromorphic reinforcement learning by training the sensor array with a weak light pulse.^[^ ^58b^ ^]^ Flexibility, high integration, and ultra‐sensitivity are essential for artificial vision systems attempting to mimic biological processing.

As a distinctive nanocarbon, 2D structural graphene and its derivatives with a thickness of one atomic layer graphene has also been used as multifunctional platforms for ocular applications, for example, implantable optoelectronic device for retinal stimulation and optical sensing.^[^ ^59^ ^]^ On account of outstanding electrical properties, graphene‐coated contact lens platform enhanced dehydration protection by monitoring the changed water evaporation rate, and further protected eyes from the damage of electromagnetic waves through dissipating electromagnetic waves in thermal radiation.^[^ ^60^ ^]^ It is noteworthy that a potential clinical implementation of graphene‐based biomaterials has been greatly restricted attributing to their potential toxicity, relatively poor water solubility, unsatisfied distribution, absorption, and targeted delivery.

Nonporous SiNPs have been developed as a promising ophthalmic carrier thanks to their charming properties embracing a stable chemical structure, large surface area to volume ratio, and ease of surface engineering. Recent observations showed that SiNPs with small sizes (5–50 nm) could permeate into corneas. Upon topical administration, SiNPs were utilized as an effective anti‐neovascularization prophylactic agent to inhibit corneal neovascularization suffering from chemical burn.^[^ ^61^ ^]^ As one of representatives among mesoporous family used in ophthalmology, MSN‐based nanosystems presented a sustained effect on suppressing corneal and retinal neovascularization in vivo by prolonging drug residency in aqueous/vitreous humor and maintaining a long‐lasting drug concentration.^[^ ^62^ ^]^ Moreover, MSNs could conjugate with various targeted motifs to improve biocompatibility and targeted effect by enhancing cellular internalization.^[^ ^63^ ^]^ However, their biodegradability in a more reliable and safer performance should be reconsidered for further ocular applications since the retaining of SiNPs in human body may lead to an undesirable cellular toxicity and long‐term health effects.^[^ ^64^ ^]^

The unique physiochemical characteristics of inorganic nanosystems, especially electronic, optical, acoustic, catalytic, and paramagnetic properties will facilitate the development of advanced theranostic nanoplatforms in ocular photothermal therapy, photodynamic therapy, computed tomography imaging, magnetic resonance imaging, etc. Determining these optimal functionalizations of inorganic nanosystems in the process of ocular theranostics is of high significance, and will occupy an irreplaceable position in the field of ocular nanomedicine. However, their metabolic activities in ocular and systemic elimination, and the cytotoxic mechanisms/effects behind individual inorganic nanosystems in vivo impede their clinical translations and commercialization.^[^ ^65^ ^]^

### 2.7 Exosome‐Based Nanomaterials

**Figure 9:**
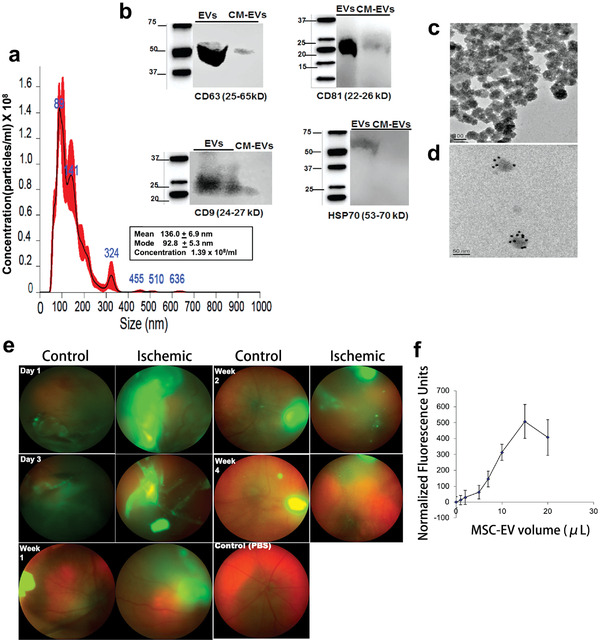
Mesenchymal stem cell (MSC)‐derived exosomes and retinal ischemia‐reperfusion. a–d) Characterization of MSC‐nanovesicles (EVs). a) Nanoparticle tracking analysis histogram showed the size distribution of isolated MSC‐EVs, and indicating that the majority of MSC‐EVs are likely exosomes. b) Western blot demonstrating the characteristic surface markers of exosomes, CD63, CD9, CD81, and HSP70*α*, expressed in MSC‐EV preparations, but not in MSC‐conditioned medium (CM) depleted of EVs. c) TEM image of cup‐shaped MSC‐EVs (approximately 100 nm) isolated from MSCs, consistent with exosomal size. d) MSC‐EVs labeled with CD63 antibody to exosome surface markers by immunogold. e) Intravitreal injection of green fluorescent MSC‐EVs into normal and ischemic eyes by fundus imaging at day 1 and 3, weeks 1, 2, and 4. Fluorescent MSC‐EVs presented for up to 4 weeks. f) Graph showed the binding of fluorescently labeled MSC‐EVs to 50 µg of isolated vitreous. The binding of MSC‐EVs to the vitreous was saturable. Data presented as mean ± SD (*n* = 6). Reproduced with permission.^[^ ^68^ ^]^ Copyright 2019, Elsevier Ltd.

Several typical nanocarriers, such as the aforementioned dendrimers and inorganic nanoparticles, have achieved considerable success by developing feasible and abundant strategies for versatile ocular applications; however, these exogenous nanosystems generated by artificial fabrication can exhibit apparent heterogeneity in various living organisms, possibly inducing potent immunogenicity and toxicity.^[^ ^64^ ^]^ In contrast, as numerous endogenous cell‐derived nanovesicles, exosomes naturally endow a low risk of immunological rejection, negligible toxicity, and superior target‐homing specificity.^[^ ^66^ ^]^ Several types of exosomes from mesenchymal stem cells (MSCs), including bone marrow, umbilical, and adipose MSCs, have been identified. Importantly, their substantial positive effects on anatomical and functional restoration of ocular tissues, such as the cornea, retina, and optic nerve, have been highlighted and are closely associated with different mechanisms, including the regulation of angiogenesis and inflammatory pathways, immunomodulation, and tissue regeneration.^[^ ^67^ ^]^ Likewise, Mathew et al. reported the neuroprotective effects of MSC‐nanovesicles, suppressing neuroinflammation and apoptosis following intravitreal administration in retinal ischemia in rats, further enhancing retinal functional recovery. Uptake of MSC‐nanovesicles by retinal ganglion cells, retinal neurons, and microglia was observed, detectable in the vitreous for 4 weeks post‐administration; this highlighted the potential of exosome‐based biomaterials to afford neuroprotection and regeneration in retinal disorders (**Figure** **9**).^[^ ^68^ ^]^ As phospholipid nanocarriers, specific small molecules or large proteins can be immersed in geometrical cytosol or inlaid on exosomal topographical lipid bilayers. Therefore, exosomes exhibit considerable potential for drug/gene delivery and immunotherapy. Upon intravitreal injection, the exosome‐mediated adeno‐associated virus was broadly targeted into the retina, suggesting that exosomes could serve as a robust nanocarrier for gene delivery and expand their application in the field of ophthalmology.^[^ ^69^ ^]^ Two clinical trials assessing exosomes, that is, exosome‐derived miRNA and MSC‐exosomes, were initiated to further explore their potential application in managing diabetic cataract and macular holes, respectively (Table 2).^[^ ^66^, ^70^ ^]^

Exosomes can be taken up by cells via four identified mechanisms, including 1) ligand‐receptor combined with cleavage and exosomal cargo release, 2) direct fusion to the cell plasma membrane and subsequent release of exosomal content into the cytoplasm, 3) receptor‐regulated endocytosis, and 4) phagocytosis, which are beneficial to better understand intercellular communication.^[^ ^71^ ^]^ However, rapid clearance from the body remains a primary drawback. Although the fate of nanovesicles administered as eye drops or via intravitreal or subconjunctival routes has not been comprehensively examined, it can be speculated that a similar rapid clearance of exosomes may also occur under some conditions. On a large scale, a sustained exosome‐based delivery platform greatly relies on producing vesicles of consistent quality and high purity, another challenge that needs to be resolved. Nevertheless, based on the above overwhelming merits, exosome‐based nanosystems afford unique advantages for the rational design of next‐generation nanomedicine, facilitating studies in the interdisciplinary field of ophthalmology and nanomedicine over the next several decades.

## 3 Fundamental Physicochemical Properties of Ocular Nanomedicine

To date, the ophthalmologic field has evidenced a range of nanoplatforms with diverse compositions and nanostructures, promoted by the unparalleled feasibility and superiority of nanotechnologies. In terms of theranostics in eye‐related diseases, determining how fundamental physicochemical properties and corresponding biological effects of ocular nanomedicine guide interactions with the surrounding environment remains a consistent theme. Bioavailability, penetration, biodistribution, and elimination of ocular nanomedicine in targeted ocular cells/tissues could be significantly affected by their differential fundamental physicochemical properties, mainly including size, hydrophilicity and/or hydrophobicity, surface charge, and biodegradability, which need to be explored to achieve optimal utilization.

### 3.1 Size

Typically, ocular nanomedicine ranges between 1 and 1000 nm, in at least one dimension. Compared with large‐scale particulate systems, corresponding ocular nanomedicines with similar chemical compositions display distinctive chemical/physical properties and biological effects.^[^ ^72^ ^]^ One overwhelming advantage of nanoparticles over their larger counterparts is improved cellular uptake. In nature, mammalian cells are generally a few microns in diameter, and their organelles appear to be considerably smaller within the nanometer range. The uptake of nanoparticles is a direct size‐dependent internalization and affords enhanced cellular uptake of smaller particles than larger particles.^[^ ^73^ ^]^ Therefore, it is postulated that nanoparticles can better locate into specific cells and organelles than micron‐sized systems. Following intraocular administration, nanoscale particles penetrate ocular physiological barriers into specific cells or tissues. Compared with larger‐scaled microspheres, considerably more fluorescence‐marked nanoscale spheres were detected in the vitreous 2 weeks after a single intravitreal delivery, with only nanospheres detected at week four following administration; this suggests the important role of biomaterial size for improved intraocular delivery.^[^ ^72^ ^]^ Considering nanoparticle biodistribution, nanoparticles with a diameter of 100–200 nm were evenly distributed in the vitreous, whereas nanoparticles at 50 nm could surpass retinal barriers and accumulate in the retina.^[^ ^17^ ^]^ Additionally, the rate constant of the in vivo circulating half‐life has received considerable attention to achieve long‐term therapeutic outcomes, which could also be impacted the biomaterial size.^[^ ^74^ ^]^ After systemic or topical administration, smaller nanoparticles are more likely excreted by the lymphatic, choroidal blood, or systemic circulation, while larger particles (preferably >100 nm) could avoid rapid clearance, allowing prolonged circulation within ocular tissues.^[^ ^21^ ^]^ These phenomena highlight that biomaterials of relatively large size are more valuable for sustained release as an ocular delivery system by prolonging their exposure and resident time in surrounding environments.^[^ ^75^ ^]^ In general, the size of ocular biomaterials is an essential parameter that determines their internalization, penetration, biodistribution, and elimination.

### 3.2 Surface Charge

From an engineering perspective, the surface charge of nanomedicines is another determinant in terms of phagocytosis, penetration, and biodistribution in ophthalmology.^[^ ^17^ ^]^ Researchers have focused on exploring the effects of electrostatic properties on “nano‐bio” interactions. Cationic nanoparticles interacting with anionic components of intercellular surfaces or membranes could result in improved particle phagocytosis, indicating that nanoparticles with a positive charge might maximize cellular internalization when compared with negatively charged counterparts.^[^ ^20^ ^]^ Interestingly, the phenomenon of biological interactions and cellular uptake in response to particle charge also demonstrates the potential for targeted delivery in the absence of specifically targeted ligands. For example, the successful delivery of therapeutic antisense oligonucleotides (ASO) or plasmid DNA by a nonbiodegradable cationic polymer has been developed, which can penetrate the cornea into the retina, enabled by electrostatic interaction with the retina.^[^ ^76^ ^]^ Furthermore, in vivo biodistribution of nanoparticles can be influenced by surface charges through electrostatic interactions with ocular tissues. In the anterior eye segment, cationic nanoparticles, such as chitosan/peptide‐decorated polymeric and triblock copolymeric micelles, can interact with the conjunctiva and cornea (with a negatively charged mucin layer) by electrical attraction, thereby prolonging drug retention and promoting nanoformulation permeability.^[^ ^25^, ^77^ ^]^ Conversely, nanoparticles with a negatively charged surface reportedly avoid adhesion to the normal or healthy ocular surface or cells, promoting tear retention time.^[^ ^78^ ^]^ In the posterior eye segments, the cellular membranes of retinal cells are characterized by negatively charged phospholipids, while the vitreous consists of a net negative charge. Considering electrostatic interactions, cationic nanoparticles are speculated to attract anionic components of the vitreous and remain localized before reaching targeted sites by delaying convective movement and diffusion, whereas anionic particles are often diffused into the vitreous body.^[^ ^79^ ^]^ Nevertheless, it is worth noting that the electrostatic explanation may not be adaptive to all types of nanomaterials considering other factors, such as stability or introduced ligands that may interfere or dynamically alter electrostatic behavior; this should be considered when further utilizing charged nanomaterials in the ophthalmologic field.

### 3.3 Hydrophilicity and/or Hydrophobicity

The inherent hydrophilic, lipophilic, or amphiphilic properties of nanomaterials exert a vital effect on permeability, endocytosis, and diffusion when nanomaterials communicate with the biological interface of different ocular structures (cornea, sclera, and choroid). In the biological interface membrane of tear film/cornea, increased hydrophilicity of the nanoparticle surface could improve drug bioavailability at the ocular surface after administration.^[^ ^22b^ ^]^ However, it has been reported that amphiphilic nanocarriers, such as peptide amphiphile nanofibers, are especially beneficial for corneal delivery, given their ability to remain at the site of interest for extended periods and the long‐term presentation of bioactive sequences.^[^ ^80^ ^]^ In contrast to relatively hydrophilic sclera alone, the trend of decreasing permeation with increasing nanoparticle hydrophobicity is remarkable in sclera‐choroid‐retinal pigmented epithelium (RPE) barriers; this indicates that the choroid‐Bruch's combination affords a more challenging obstacle for lipophilic nanomaterials when compared with hydrophilic ones.^[^ ^81^ ^]^ To achieve non‐invasive drug delivery into intraocular tissues/cells, amphiphilic core‐shell‐based nanocarriers comprising hydrophobic cores (e.g., polycaprolactone, to encapsulate hydrophobic drugs) and a hydrophilic shell (e.g., PEG, to provide excellent water dispersity) have been proposed. These strategies exhibit significant potential for the treatment of posterior segment ocular diseases by topical administration.^[^ ^82^ ^]^ Additionally, the hydrophilicity and/or hydrophobicity of nanomaterials is considered a critical aspect affecting cellular internalization by accommodating cellular membrane wrapping processes. The underlying basis of biological interactions with cells depends on the hydrophobic nature of nanoparticles, wherein nanosystems with more hydrophobic components than those in cellular membranes allow enhanced endocytosis and substrate combination. Conversely, nanomaterials with hydrophilic surfaces can undergo optimal uptake by retinal cells.^[^ ^83^ ^]^ Therefore, for ocular delivery, nanomaterials exhibiting hydrophilic, lipophilic, or amphiphilic properties need to be carefully considered under specific conditions prior to application.

### 3.4 Biodegradability

**Figure 10:**
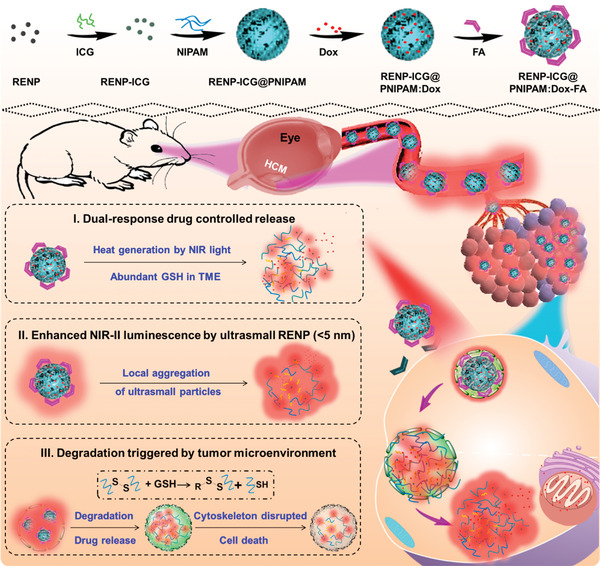
Schematic illustration of the fabrication and application of dual‐response nanocomposites in chemophotothermal therapy for choroidal melanoma, and the microenvironment‐triggered degradation of cross‐linked poly(nisopropylacrylamide) (PNIPAM) hydrogels. RENP, rare‐earth nanoparticles; ICG, indocyanine green; Dox, doxorubicin; FA, folic acid; HCM, human choroidal melanoma; GSH, glutathione; NIR, near‐infrared window; TME, tumor microenvironment. Reproduced with permission.^[^ ^85^ ^]^ Copyright 2020, American Chemical Society.

Currently, numerous degradable nanoparticles have achieved improved ocular drug delivery, possibly attributed to their positive effects on prolonging cargo release and retention, along with alleviated cytotoxicity in ocular cells/tissues. The bioavailability and therapeutic window of cargo released from biodegradable ocular nanomedicine are strongly determined by the degradation rate of carriers in the biological milieu, and the degradation rate of nanomedicine greatly depends on their composition, molecular weight, and route of administration.^[^ ^84^ ^]^ For instance, nanomaterials with a low molecular weight typically undergo rapid degradation, that is, instability tends to disassemble or aggregate, which could cause burst release of cargo and compromised therapeutic outcomes. However, high‐molecular‐weight biomaterials will undergo slow in vivo degradation, inducing the risk of ocular tissue accumulation. Therefore, a controllable degradation rate for ocular nanomedicine in regions of interest remains critical to obtain higher drug availability without cytotoxicity. Accordingly, Li et al. designed microenvironment‐triggered degradable hydrogels based on ultrasmall rare‐earth nanoparticles with enhanced NIR‐II luminescence, capable of drug release by responding to glutathione and heat energy in the tumor microenvironment. The degradability of the hydrogel composites under physiological conditions is conducive for alleviating long‐term biological toxicity and promoting a photothermal‐chemotherapeutic effect (**Figure** **10**).^[^ ^85^ ^]^ Conversely, the biodegradability of nanomedicine remains a particularly important consideration in gene‐based therapies for repeated gene transfection.^[^ ^86^ ^]^ Following cell uptake, nanocarriers need to escape intracellular compartments, such as endosomes and lysosomes, to sequentially unpack their gene payloads, such as pDNA and siRNA, via degradation.^[^ ^87^ ^]^ Nanocarriers possessing non‐degradable bonds and high molecular weight may markedly accumulate in normal cells, resulting in adverse effects on the metabolic activities and transportation of normal cells, eventually causing high cytotoxicity, especially after repeated administration.^[^ ^88^ ^]^ Thus, nanocarriers with biodegradable features benefit gene delivery processes by effectively unpacking the loaded therapeutic genes, reducing accumulation, and alleviating toxicity in targeted cells. Currently, the biodegradability of nanomaterials is the most critical feature that impacts the clinical applicability of corresponding final biomedical products, given their substantial influence on biocompatibility and biosafety. Despite exhibiting favorable biodegradability and biocompatibility, the development of biodegradable ocular nanomaterials in clinical trials remains an immense challenge.

In summary, ocular nanomedicine has undergone tremendous growth based on a gradual understanding of how these parameters collectively influence the “nano‐bio” interface, especially in terms of endocytosis, penetration, cargo release, biodistribution, and elimination after interacting with ocular surroundings. Among the four key factors, the size of ocular nanomedicine plays an essential role in improving cellular uptake and penetration across ocular physiological barriers into ocular cells/tissues. Surface charge significantly influences in vivo biodistribution of nanoparticles by electrostatic interaction with intended ocular tissues. In addition, hydrophilicity and/or hydrophobicity can critically impact permeability and diffusion across biological interfaces of different ocular structures, whereas biodegradability markedly influences biosafety, cargo release, and retention in intended ocular cells. Therefore, the value of optimizing and tailoring these parameters of ocular nanomedicine to improve therapeutic outcomes needs to be highlighted in future applications.

## 4 Surface Engineering of Ocular Nanomedicine

Unmodified ocular nanomedicines with unique structure/composition and natural physicochemical configurations can enhance ocular penetration and bioavailability and allow sophisticated kinetic release. However, personalized ocular medicine, including controllable and targeted release, on‐demand gene delivery, pathology‐oriented diagnostics and therapeutics (theranostic), and side‐effect mitigation for specific paradigms, remains a challenge. For instance, actively targeting pathological regions by binding targeted agents to nanomedicine is necessary to achieve the high efficacy of synergistic therapy in ophthalmology. Accordingly, the functionalization of ocular nanomedicine with programmable properties using surface engineering with one or more additional entities can provide unprecedented opportunities to meet these requirements. Presently, three specific surface engineering approaches in ocular nanomedicine include: 1) decorating surfaces with certain molecules/polymers, 2) targeted modification of functional ligands/motifs, and 3) loading various therapeutic substances.

Considering the first approach, that is, decorating ocular nanomedicine with a certain molecule, such as amino and carboxylic acids, or polymers (such as PEG and chitosan), has been employed to improve the physiological stability and bioavailability by controllable and targeted properties.^[^ ^10^, ^79^ ^]^ Amino‐functionalized MSNs as nanocarriers for drug delivery achieved sustained drug release over 8 h, enhancing ocular bioavailability and allowing mucoadhesion owing to the presence of hydroxyl and amino groups.^[^ ^89^ ^]^ Coating with an immune evasion PEG polymer prevented recognition and uptake of ocular nanomedicine by metabolic organs in the host, with subsequent removal by macrophages in vivo. This strategy significantly improves the bioavailability of nanomaterials, thus contributing to compromised clearance and prolonging blood circulation and retention time prior to the second dose.^[^ ^90^ ^]^ In addition to polymeric PEG decoration, nanocarriers are directly modified with a mucoadhesive molecule, such as chitosan, to functionalize the sulfobutylether‐*β*‐cyclodextrin nanoparticles, tremendously enhancing the ocular controlled drug release with a high retention time and permeability across the ocular surface.^[^ ^77a^ ^]^

The second approach for surface engineering in ocular nanomedicine is targeted modification of functional ligands/motifs to accomplish site‐specific therapy.^[^ ^84b^ ^]^ The successful development of antibody technologies has increased the specificity of ocular nanomedicine via bioconjugation with different affinity ligands, ranging from biomaterials (e.g., HA), peptides (e.g., RGD peptide), and proteins (e.g., transferrin) to nucleic acids (aptamers or oligonucleotides). For example, high expression of the CD44 receptor on ocular cells, such as Müller cells and the RPE layer, could combine with HA by receptor‐mediated endocytosis, resulting in site‐ and time‐specific drug delivery to posterior ocular tissues.^[^ ^10a^ ^]^ Typically, functionalized QDs with *α*v*β*3 integrin receptor‐specific cyclo peptide significantly improved the combination and cellular uptake in retinal capillary endothelial cells, substantially upregulating the expression of *α*v*β*3 integrin receptor in the early phase of degeneration.^[^ ^91^ ^]^ Therefore, ocular nanomedicine mediated by specific receptor‐targeted ligands would establish a basis for preventive interventions for ocular disorders at an early stage.^[^ ^49b^ ^]^ Notably, various characteristics of ligand molecules, including solubility, biodistribution, target tissue accumulation, plasma binding, and elimination after incorporation into ocular nanomedicine, should not be restricted by the chemical compositions of nanosystems and should become an in‐part function of physicochemical properties in ocular nanomedicine.

**Figure 11:**
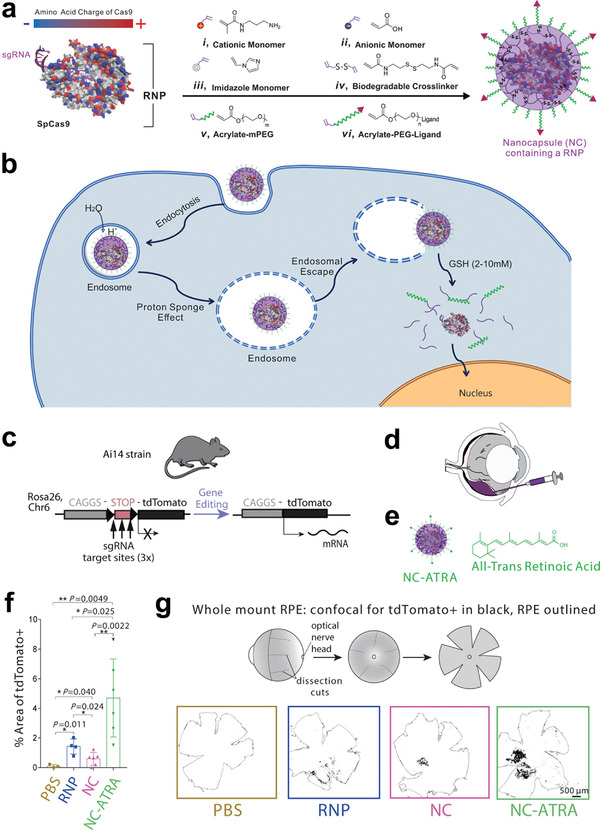
A biodegradable nanocapsule delivers a CRISPR‐Cas9 ribonucleoprotein (RNP) complex for in vivo genome editing. a) *Sp*.Cas9 showed a heterogeneous surface charge due to both negative and positive amino acids residues, as well as the negatively charged sgRNA. A schematic depiction for the formation of covalently crosslinked, yet intracellularly biodegradable, nanocapsule to deliver RNP complex by in situ free radical polymerization. b) A schematic illustration of the proposed mechanism of cellular uptake of nanocapsule and subcellular release of RNP. c) Schematic illustration of tdTomato locus within the Ai14 mouse strain. A STOP cassette including three SV40 polyA sequences prevented the transcription of downstream red fluorescent protein variant, tdTomato (left). When cells are edited by CRISPR/Cas9 to excise the STOP cassette via cut sites present in each of repeats, they will express tdTomato (right). d) Scheme of the targeted genome editors in retinal pigmented epithelium (RPE) cells by subretinal injection. e) Illustration of nanocapsule‐ATRA. f) The efficiency of genome editing as quantified by percent of area of whole RPE with genome editing reporter (tdTomato+). PBS (*n* = 3), RNP (*n* = 4), NC (*n* = 6), or NC‐ATRA (*n* = 6). Data presented as mean ± SD. g) Scheme of whole mount RPE preparation and representative photos of tdTomato+ signal (black) after subretinal injection for 12 days. Whole RPE layer is outlined. *n* = 3. Reproduced with permission.^[^ ^94^ ^]^ Copyright 2019, Nature Publishing Group.

The third surface engineering strategy involves encapsulating therapeutic substances, including small molecules, peptides, proteins, nucleic acids, and clinically available biopharmaceuticals, which are then released at targeted pathological sites to accomplish corresponding diagnostic and therapeutic functions. For instance, MSNs used to deliver sodium nitroprusside (a small molecular nitric oxide‐donating drug) readily overcame drawbacks such as short duration of action and poor corneal penetration by delivering the drug to the targeted Schlemm's canal and trabecular meshwork.^[^ ^92^ ^]^ Typically, bioactive macromolecules, such as nucleic acids (DNA, siRNA, or miRNA), could be a highly efficient host bioreactor to code functional proteins for treating eye‐related diseases; however, they demonstrate relatively poor penetration into desired subcellular compartments, such as endosomal membranes. Thus, nanocarriers must escape from lysosomes prior to endosomal fusion.^[^ ^93^ ^]^ Additionally, following topical administration, the delivery of large and unstable nucleic acids into intraocular tissues could be significantly obstructed by inherent ocular absorption barriers. Alternatively, cationic liposomal components or polymers can be employed to encapsulate multiple CRISPR components into large particles (typically >100 nm in diameter). Chen et al. designed a customizable nanocapsule (25 nm diameter) based on a thin glutathione‐cleavable covalently cross‐linked polymer, coating around a preassembled ribonucleoprotein (RNP) complex between a Cas9 nuclease and a single guide RNA (sgRNA). This customizable nanocapsule efficiently generated robust, targeted gene editing without apparent cytotoxicity, affording in situ delivery of CRISPR RNP complexes (**Figure** **11**).^[^ ^94^ ^]^ Therefore, safe and effective non‐viral vehicles using different surface engineering methods are promising for ocular on‐demand gene delivery.

Collectively, targeted strategies associated with ocular nanomedicine predominantly involve passive and active approaches. The passive targeted strategy (bearing no specific ligands) is directly related to their inherent physicochemical properties (e.g., size, charge), whereas the active targeted strategy is functionalized with specific affinity ligands (therapeutic or imaging agents) to facilitate cellular internalization in a highly specific manner. Additionally, to fulfill the theranostic properties of ocular nanomedicine by surface engineering with programmable entities, several factors should be carefully considered: 1) careful selection of conjugated chemical moieties to avoid unexpected toxicity resulting from residues during the synthetic process; 2) maintaining functional chemical components in ocular nanomedicine to prevent destruction by modified cargo; and 3) on‐demand release of encapsulated substances from the backbone of ocular nanomedicine while retaining functional and therapeutic properties. These proposed strategies will form a basis to achieve personalized ocular medicine in specific paradigms.

## 5 Applications of Ocular Nanomedicine

Ocular diseases exert a direct impact on visual function and affect the quality of human life. However, most available strategies for targeting ocular diseases exhibit compromised therapeutic efficacy. Over the past several decades, nanotechnology, by employing sophisticated methodologies, has overtly improved the therapeutic effectiveness of ocular nanomedicine by modulating particle structures/compositions and fundamental physicochemical properties or further functionalization using surface engineering to fulfill multiple theranostic applications. The pathogenic‐oriented principle of ocular nanomedicine is an evolutionary concept that greatly depends on whether different pathogenesis involved in ocular diseases can be targeted to attain an effective therapeutic window, as susceptibility often decreases with time. The most prevalent pathological processes in various ocular diseases have been identified and are mainly classified into inflammatory (e.g., uveitis, diabetic cataracts, and dry AMD), elevated IOP (e.g., glaucoma), bacterial infection (e.g., bacterial keratitis and endophthalmitis), neovascularization (e.g., corneal neovascularization and wet AMD), tumors (e.g., retinoblastoma and uveal melanoma), and refractive error (e.g., myopia). Therefore, numerous ocular nanomedicine‐assisted strategies have been investigated to promote ocular therapeutic outcomes based on the pathogenic‐oriented principle (**Table** **1**).

**Table 1:** Summary of representative ocular nanomedicine for ocular disease treatment

Nanoformulations	Cargos	Administrations	Functions/Applications	Reference
Liposomes	CRISPR‐Cas9 ribonucleoprotein complex	Subretinal injection	Inducing efficient genome editing in vivo	^[^ ^94^ ^]^
Nanostructured lipid carriers	Dasatinib	Topical administration	Increasing the solubility of dasatinib, sustaining drug release, reducing ocular toxicity, and facilitating its penetration into cornea	^[^ ^23b^ ^]^
Solid lipid nanoparticles	RS1 DNA plasmid	Intravitreal injection	Improvement of retinal structure by extending therapeutic period up to 2 weeks	^[^ ^22a^ ^]^
DSPE‐PEG_2000_‐cRGD nanomicelle	Flurbiprofen	Eye drops	Specifically targeted combining to integrin receptors on cornea surface and facilitated rapid mucoadhesion	^[^ ^28^ ^]^
Boronic acid‐rich dendrimer	Superoxide dismutase	Intravitreal injection	Protecting retinal function and reducing cell apoptosis by high levels of cellular uptake without immunogenicity and cytotoxicity	^[^ ^33^ ^]^
DNA/PLGA hybrid hydrogel	Dexamethasone	Topical administration	Improving dexamethasone accumulation and mediating gradual dexamethasone release	^[^ ^4^ ^]^
Chitosan‐based self‐assembly nanofilms	Heparin	Cornea in situ self‐assembly	Rescuing corneal defective wound healing	^[^ ^45^ ^]^
Mesenchymal stem cells‐derived exosomes	–	Intravitreal injection/eye drops	Significantly anatomical and functional restoration of retina, optic nerve, or cornea by modulating angiogenesis and inflammation, and improving immunomodulation and tissue regeneration	^[^ ^66^, ^67^, ^68^ ^]^
Palladium‐coated gold nanorods	–	Being pasted to lacrimal gland	Sensing diverse visible light irradiations and spontaneously responded by heating up to secrete more tears to prevent dry eye	^[^ ^50^ ^]^
Nanoceria	Doxorubicin	Intravitreal injection	Sensitive to extracellular acidic pH conditions, targeted to tumor cells, instantaneously inducing ROS, and releasing doxorubicin intracellularly to enhance the chemotherapeutic activity in retinoblastoma cells	^[^ ^122a^ ^]^
Gold nanoparticles‐based nanobubbles	Hyaluronic acid	Intravitreal injection	Mechanically ablating the vitreous opacities upon local pulsed‐laser illuminates to generate vapor nanobubbles	^[^ ^51^ ^]^
Dichalcogenides quantum dots	—	Eye drops	Antibacterial agent with a fast response and no reliance on light	^[^ ^57^ ^]^
Mesoporous silica nanoparticles	Bevacizumab	Intravitreal/subconjunctival injection	Suppressing corneal and retinal neovascularization	^[^ ^62^ ^]^
Spermidine‐derived carbon quantum dots	—	Eye drops	Strong antibacterial capabilities for treatment of bacterial keratitis	^[^ ^10c^ ^]^
Graphene‐coated contact lens	—	Wearing on the ocular surface	Monitoring changed water evaporation rate and protecting eyes from electromagnetic wave damage	^[^ ^59^, ^60^ ^]^
Thermosensitive triblock copolymer	Acriflavine hydrochloride and sunitinib malate	Topical administration	Increasing the intraocular absorption of hydrophilic and hydrophobic drugs and extending the drug–ocular–epithelium contact time	^[^ ^140^ ^]^
PEG‐*b*‐poly(propylene sulfide) nanomicelles	Actin depolymerizer latrunculin A	Intracameral injection	Selectively modulating stiffness of Schlemm's canal cells for therapeutic reducing intraocular pressure	^[^ ^105^ ^]^

### 5.1 Ocular Inflammation/Oxidative‐Stress Therapy

**Figure 12:**
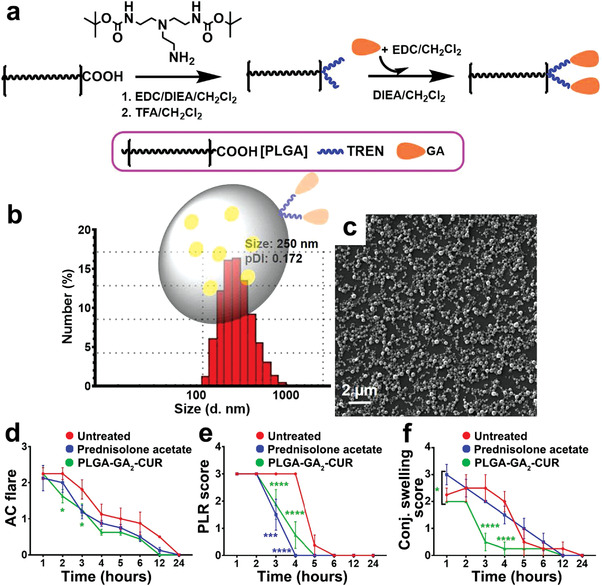
Synthesis and anti‐inflammatory effects of curcumin (CUR)‐loaded gambogic acid (GA)–coupled‐PLGA (PLGA‐GA_2_) nanoparticles (PLGA‐GA_2_‐CUR). a) Schematic illustration of the synthesis of PLGA‐GA_2_‐CUR. b) Characterization of PLGA‐GA_2_‐CUR by the dynamic light scattering size distribution with an insert depicting model particle. c) Scanning electron microscope (SEM) image of PLGA‐GA_2_‐CUR. d–f) Anti‐inflammatory effects of topical PLGA‐GA_2_‐CUR on a canine model with acute intraocular inflammation. After intracameral administration of lens protein at *t* = 0 h, eyes were serially analyzed by the semiquantitative preclinical ocular toxicology scoring (SPOTS) system, including evaluation of d) aqueous flare, e) pupillary light reflex, and f) conjunctival swelling. In contrast to topical treatment with prednisolone acetate and untreated controls, there was a significance of topical PLA‐GA_2_‐CUR injection as determined by a two‐way ANOVA. *p < 0.05, **p < 0.01, ***p < 0.001, and ****p < 0.0001. TFA, trifluoroacetic acid; TREN, tris(2‐aminoethyl)amine; DIEA, *N,N*‐diisopropylethylamine; EDC, 1‐ethyl‐3‐ (dimethylaminopropyl) carbodiimide. Reproduced with permission.^[^ ^98^ ^]^ Copyright 2020, AAAS.

Human eyes are considered to be sealed from systematic circulation, and the retina is an “immune‐privileged” zone.^[^ ^56^ ^]^ Therefore, inflammation in the eye plays a pathological role in a series of ocular diseases, including uveitis, diabetic cataracts, and dry AMD, by disrupting phospholipids in the cell membrane and excessive release of inflammatory substances such as prostaglandins. These harmful substances could induce inflammatory ocular disease, accompanied by typical clinical symptoms such as photophobia, opacification, pain, posterior capsule, vasodilatation, increased vascular permeability, and IOP. For example, uveitis is a typical ocular inflammatory disorder in the uvea, possibly induced by virus/bacterial infection or a particulate matter‐induced over‐reactive immune system response.^[^ ^95^ ^]^ Dexamethasone, corticosteroid, and triamcinolone acetonide exert anti‐inflammatory activity for treating uveitis; however, long‐term release at the targeted site remains an immense challenge.^[^ ^96^ ^]^ Alternatively, the controllable release of drugs by biodegradable nanocarriers, such as PLGA‐based nanoparticles, has been successfully achieved with sustained anti‐inflammatory drug release in an experimental autoimmune uveitis animal model, thereby suppressing ocular inflammation without complications.^[^ ^97^ ^]^ More recently, Ganugula et al. designed the receptor‐mediated delivery of curcumin (CUR) assisted by double‐headed gambogic acid (GA)‐coupled PLGA, that is, PLGA‐GA_2_‐CUR nanoparticles. Oral administration of these nanoparticles in a canine model with lens‐induced uveitis showed a notable drug level in aqueous humor and afforded protection against intraocular inflammation with reduced miosis, aqueous flare, and chemosis in the acute phase (<4 h) when compared with commercial anti‐inflammatory treatment (oral carprofen) (**Figure** **12**).^[^ ^98^ ^]^

Excessive ROS is undoubtedly involved in the pathological origin of abnormal inflammatory responses during ocular inflammatory disorders, especially diabetic cataracts and AMD. Diabetic cataract is an ocular complication in patients with diabetes who often suffer from diminished visual function, and AMD is the leading cause of vision loss in elderly individuals with progressive photoreceptor death.^[^ ^99^ ^]^ However, currently available drugs demonstrate limited ability to effectively delay and prevent diabetic cataracts and dry AMD. Several studies have reported that oxidative damage in the crystalline lens and photoreceptor also plays a crucial role in the pathogenesis of diabetic cataract and dry AMD, respectively. Thus, reducing ROS levels could potentially alleviate inflammation‐associated impairment.^[^ ^100^ ^]^ Currently, two classes of work mechanisms have been investigated in ROS‐mediated inflammatory ocular diseases: 1) endowing ROS‐scavenging ability for guiding detoxification or 2) imparting ROS‐response anti‐inflammatory drug release.^[^ ^101^ ^]^ However, conventional antioxidants and anti‐inflammatory biopharmaceuticals remain largely inefficacious in ophthalmology, given their poor permeability, nonspecific biodistribution, low retention at pathological sites, and rapid renal excretion. Depending on either the non‐enzymatic or catalytic features, it has been well documented that autoregenerative redox nanoceria can effectively protect lens epithelial cells and photoreceptors from oxidative damage based on the progressive elimination of ROS, thereby maintaining lens transparency in diabetic cataracts and delaying photoreceptor degeneration in AMD for a prolonged period.^[^ ^99a^ ^]^

Interestingly, INVELTYS (Kala Pharmaceuticals), bromfenac DuraSite, and DexaSite are important ocular nanomedicines that have succeeded in clinical trials and/or obtained FDA approval for treating postoperative inflammation and pain following ocular surgery (**Table** **2**). INVELTYS, as a KPI‐121 1% ophthalmic nanosuspension of loteprednol etabonate, is delivered as mucus‐penetrating particles coated with low molecular weight PEG. Primary outcomes in clinical trials revealed that INVELTYS was safe and effectively resolved postoperative ocular inflammation and subject‐rated ocular pain following cataract surgery when administered twice daily for 2 weeks. The observed results could be attributed to mucus‐penetrating particles that allow the drug to efficiently penetrate the mucin layer of the tear film, facilitating drug release to underlying ocular tissues.^[^ ^102^ ^]^

**Table 2:** Representative ocular nanaomedicine in clinical trials and/or obtained FDA approval for ocular disease treatment

Products/nanoformulations (cargos)	Conditions/diseases	Administrations	Number enrolled	Status	Phase	ClinicalTrials.gov Identifier
Nanoparticles (urea)	Cataract	Eye drops	50	Completed	II	NCT03001466
Albumin (paclitaxel)	Melanoma	Intravitreal injection	4	Completed	II	NCT00738361
Cyclodextrin (dexamethasone)	Diabetic macular edema	Eye drops	40	Unknown^a)^	II/III	NCT01523314
Lecithin/glycerin (coenzyme Q10)	Ataxia with ocular apraxia type 1	Oral	19	Completed	III	NCT02333305
Liposome (latanoprost)	Ocular hypertension	Subconjunctival injection	6	Completed	I/II	NCT01987323
Liposomes (latanoprost)	Ocular hypertension and open‐angle glaucoma	Subconjunctival injection	81	Completed	II	NCT02466399
Liposomes (vincristine)	Metastatic malignant uveal melanoma	Intravitreal injection	54	Completed	II	NCT00506142
Liposomes (vincristine)	Retinoblastoma	Intravitreal injection	331	Unknown^a)^	III	NCT00335738
Lipid (TLC399)	Retinal vein occlusion, macula edema	Intravitreal injection	61	Active, not recruiting	II	NCT03093701
Lipid (TLC399)	Central/branch retinal vein occlusion with macula edema	Intravitreal injection	30	Recruiting	I/II	NCT02006147
INVELTYS/Mucus penetrating particles (Loteprednol etabonate)	Postoperative inflammation and pain following ocular surgery	Topical administration	900	Completed	FDA approved	NCT02163824 and NCT02793817
Bromfenac DuraSite/synthetic polymer of cross‐linked polyacrylic acid (bromfenac)	Post cataract surgery inflammation and pain	Topical administration	268	Completed	FDA approved	NCT01576952
DexaSite/Synthetic polymer of crosslinked polyacrylic acid and chitosan (dexamethasone)	Post cataract surgery inflammation and pain	Topical administration	260	Completed	Phase III	NCT03192137
Cyclosporine nanomicellar	Dry eye	Eye drops	258	Completed	III	NCT02845674
Cyclosporine nanomicellar	Dry eye	Eye drops	745	Completed	III	NCT02688556
Mesenchymal stem cells‐derived exosomes	Macular holes	Intravitreal injection	44	Recruiting	Early Phase I	NCT03437759
Serum exosomal miRNA	Diabetic retinopathy	—	200	Not yet recruiting	I	NCT03264976

^a)^ Study has passed its completion date and status has not been verified in more than 2 years.

### 5.2 Ocular Hypertension Therapy

**Figure 13:**
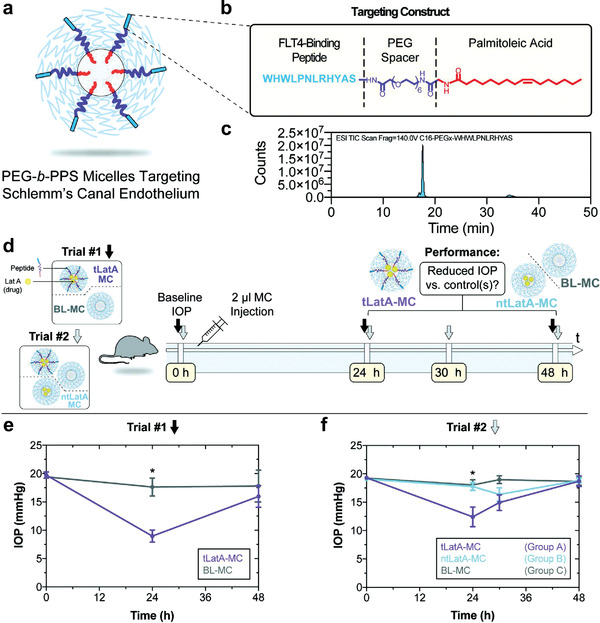
Targeted delivery of self‐assembled poly (ethylene glycol)‐*b*‐poly(propylene sulfide) (PEG‐*b*‐PPS) micelles (MC) to Schlemm's canal endothelial cells for glaucoma treatment. a) Schematic illustration of peptide‐displaying micelles. b) The peptide‐targeting construct including PEG spacer, targeting peptide, and palmitoleic acid tail. c) LCMS spectra of the purified Flt4‐targeting peptide construct. d–f) Targeted delivery of latrunculin in mouse eyes. d) Illustrative overview of two different intraocular pressure (IOP) measurement schedules. Trials #1 and Trial #2 IOP timepoints presented with black and gray arrows, respectively. e) The Trial #1 consisted of 2 µL intracameral injection of black (BL)‐MC or tLatA MC (40 mg mL^−1^, 5% peptide, 17 µM LatA). IOP was detected prior to injection, and after 24 and 48 h. f) In trial #2, 2 µL of BL‐MC, ntLatA‐MC, or tLatA‐MC (15.5 µM LatA, 40 mg mL^−1^ 5% peptide) micelles were injected into one eye of 5 mice each. IOP was detected prior to injection and at three time points during a 48 h time course. Data presented as mean ± SEM (*n* = 5). Trial #1 and # 2 significance determined by unpaired *t*‐test and ANOVA with post hoc Tukey's multiple comparisons test (*p < 0.03), respectively. Reproduced with permission.^[^ ^105^ ^]^ Copyright 2020, Wiley‐VCH.

Glaucoma, a type of optic neuropathy resulting from the damaged optic nerve, affects more than 60 million people worldwide.^[^ ^103^ ^]^ Several pathogenic parameters have been implicated in glaucoma progression, including elevated IOP, optic nerve ischemia, and oxidative stress‐related activation.^[^ ^2a^ ^]^ Although an elevated IOP is not the only factor attributed to glaucoma development, reducing the elevated IOP directly affords effective neuroprotection and is a primary modifiable target for glaucoma control from a pathophysiological perspective.^[^ ^103^ ^]^ Daily topical drug application as eye drops to decrease the elevated IOP and oxidative stress is one therapeutic strategy employed in patients with glaucoma. However, developing a product for long‐term medication using eye drops remains a potential challenge in chronic glaucoma. Nanotechnology has been used as a valuable tool to fabricate different nanosystems, such as lipid DNA nanoparticles, hollow ceria nanoparticles, and layer‐by‐layer coated siRNA nanoparticles. Reportedly, nanoparticles could afford long‐term alleviation of glaucomatous damage without disrupting the vesicle shape, owing to potent inherent antioxidant and anti‐inflammatory functions or controllable and sustained release of anti‐IOP drugs or siRNA gene silencing.^[^ ^26^, ^104^ ^]^ More recently, a cell‐softening nanotherapy has been proposed to reduce IOP by targeted binding to the vascular endothelial growth factor receptor 3 (VEGFR3)/FLT4 receptor expressed in Schlemm's canal cells. These nanomicelles (tLatA‐MC) are based on a targeted PEG‐*b*‐poly(propylene sulfide) micelle (PEG‐*b*‐PPS) and loaded with the actin depolymerizer latrunculin A (tLatA) to selectively modulate the stiffness of Schlemm's canal cells (an important pathophysiology during glaucoma). tLatA‐MC reduced IOP in a mouse model by 30–50%, indicating the functional efficacy of tLatA‐MC for glaucoma treatment (**Figure** **13**).^[^ ^105^ ^]^ Conversely, a non‐invasive approach using nanotechnology to monitor the stiffness of the trabecular meshwork in vivo also exhibits significant potential to improve the clinical care of glaucoma.^[^ ^106^ ^]^ These promising results notably demonstrate the enormous promise of an ocular nanomedicine‐based strategy for chronic glaucoma therapy.

### 5.3 Ocular Bacterial Infection Therapy

Bacteria often play a significant antagonistic and pathogenic role in self‐contained and immune‐privileged ocular tissues by disrupting normal metabolic processes and even causing cell death, collectively considered ocular bacterial infections.^[^ ^56^ ^]^ The downstream diseases of ocular bacterial infections in the external (e.g., bacterial keratitis) and inner parts of the eyes (e.g., endophthalmitis) may pose a serious threat to visual health. Therefore, it is crucial to employ efficacious therapeutic interventions to address this dilemma. Over several decades, antibiotics have rapidly advanced as ideal terminators of nearly all bacteria‐mediated diseases. However, this expectation has been quickly tempered with the emergence of the first‐rank bacterial pathogens, that is, multidrug‐resistant (MDR) bacteria, particularly *Escherichia coli* and *Klebsiella pneumoniae*, which mainly contribute to the existence of biofilms.^[^ ^107^ ^]^ Biofilms are reportedly involved in 80% of bacterial infectious diseases and are composed of extracellular polymeric substances, water, and bacteria. Among them, bacteria play a vital role in protecting biofilm matrices by markedly increasing the resistance to bactericides. For instance, the dormancy of the bottom bacteria in MDR bacteria significantly undermined bactericide permeability. Accordingly, versatile antibacterial surface/antibiofilm‐based ocular nanomedicines have been developed to kill live bacteria and remove mature biofilms or decrease bacterial adhesion to suppress biofilm development, resulting in potent antibacterial treatment.

**Figure 14:**
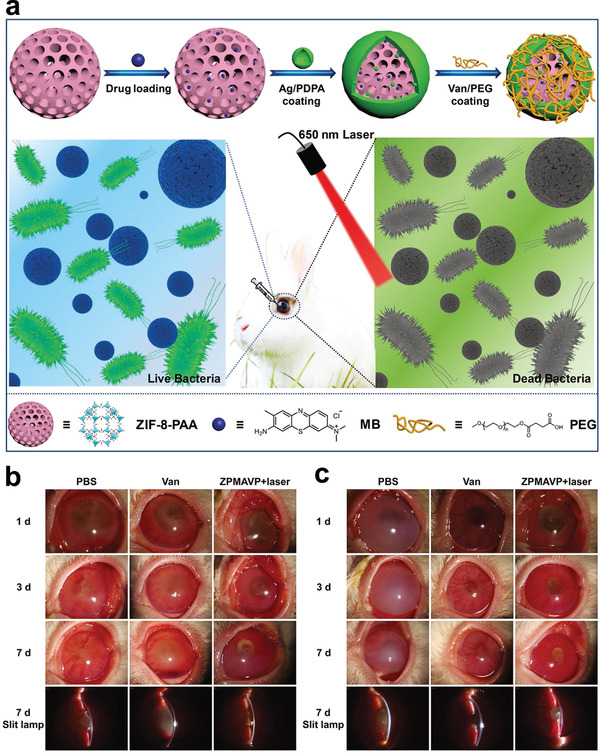
A distinctive hybrid system based on zeolitic imidazolate framework (ZIF) for synergistic photodynamic therapy and chemotherapy of endophthalmitis. a) Schematic illustration of constructed hybrid system ZIF‐8‐polyacrylic acid (ZIF‐8‐PAA)‐methylbenzene blue (MB)@silver nanoparticles (AgNPs)@vancomycin (Van)/NH‐PEG (ZPMAVP) for endophthalmitis treatment by synergistic chemotherapy and photodynamic therapy. b,c) Photographs and slit lamp micrographs of endophthalmitis resulted from *Staphylococcus aureus* (b) and methicillin‐resistant staphylococcus aureus (MRSA) (c) after treatment with three groups of PBS, Van, and ZPMAVP nanoparticles + laser (202 mW cm^−2^) at 1, 3, and 7 days, respectively. Reproduced with permission.^[^ ^112^ ^]^ Copyright 2019, Wiley‐VCH.

Patients with bacterial keratitis typically experience unexpected corneal epithelium damage, owing to difficulties in spontaneous recovery after bacterial infection. Therefore, it is crucial to develop an effective approach to eradicate MDR infections and simultaneously promote injury healing.^[^ ^52a^ ^]^ More recently, Qiao et al. proposed a composite cupriferous hollow nanoshell (AuAgCu2O NS) comprising a Cu_2_O shell and a hollow gold‐silver (AuAg) core as a high‐quality photothermal therapeutic agent for treating non‐healing keratitis. The authors revealed that Ag released from the hollow AuAg core exhibited a synergistic effect against MDR bacteria. In addition, copper ions released from the Cu_2_O shell hastened fibroblast cell migration and endothelial cell angiogenesis, augmenting the wound healing efficacy in keratitis models.^[^ ^108^ ^]^ Endophthalmitis is primarily caused by pathogenic microorganisms, particularly bacteria, which can lead to eyeball damage and distinct inflammation in ocular tissues, especially the uvea and vitreous cavity, seriously threatening the visual function of patients.^[^ ^109^ ^]^ Currently, comprehensive elimination of endophthalmitis remains a challenge, and clinical antibiotics are only employed to control progressive development.^[^ ^110^ ^]^ Thus, a strategic approach using targeted phototherapy has been proposed to manage endophthalmitis by biofilm eradication.^[^ ^111^ ^]^ For example, synergistic photodynamic therapy and chemotherapy to treat endophthalmitis were developed using a pH‐responsive hybrid system, that is, zeolitic imidazolate framework‐8‐polyacrylic acid (ZIF‐8‐PAA)‐methylbenzene blue (MB)@silver nanoparticles (AgNPs)@vancomycin (Van)/NH‐PEG (ZPMAVP). ZIF‐8‐PAA significantly improved the drug‐encapsulation capability, facilitated controllable and targeted delivery of a photosensitizer antibacterial agent MB, and reduced AgNO into AgNPs in situ by dopamine modification. Upon secondary functionalization with Van/NH‐PEG, this platform ultimately formed a synergistic composite. Generally, nanomaterials can rapidly release MB to enrich the local therapeutic concentration and damage bacterial biofilms with good biocompatibility, suggesting the superiority of synergistic photodynamic therapy and chemotherapy over any single therapeutic strategy for bacterial ophthalmic diseases (**Figure** **14**).^[^ ^112^ ^]^

### 5.4 Ocular Neovascularization Therapy

**Figure 15:**
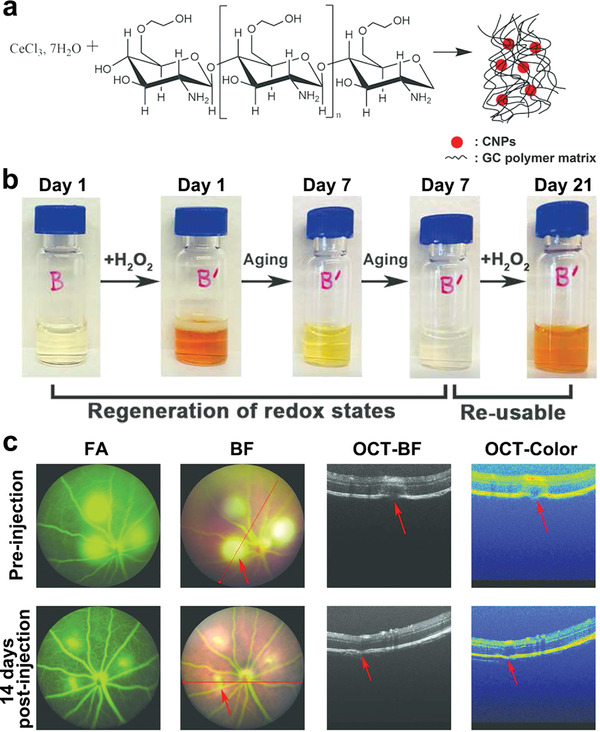
Auto‐regenerative antioxidant significantly attenuates choroidal neovascularization. a) Schematic illustration of glycol chitosan‐coated antioxidant ceria nanoparticles (GCCNP) synthesis. b) Photos of color changes of GCCNPs on addition of H_2_O_2_, reflecting auto‐regenerative nature of GCCNPs. c) Representative fundus (fluorescein angiography (FA) and BF) and optical coherence tomography (OCT) photographs (OCT‐BF and OCT‐color) before and 14 d following intravitreal injections of GCCNPs after laser induction. Red arrows represented the laser‐damaged areas. Reproduced with permission.^[^ ^99b^ ^]^ Copyright 2017, American Chemical Society.

Ocular neovascularization, particularly corneal neovascularization and CNV, is one primary pathological phenotype known to occur in corneal and retinal diseases, respectively. The normal cornea is avascular, and corneal neovascularization is often initiated by ocular surface injuries that can disrupt the harmonic balance between angiogenic inhibitors and stimulators, eventually resulting in visual impairment.^[^ ^80^ ^]^ In wet AMD, pathogenic vessels protrude through Bruch's membrane and then grow into the subretinal space, leading to initial CNV and distorted central vision.^[^ ^113^ ^]^ For antiangiogenic therapeutics, targeted inhibition of VEGF (a predominant stimulator during angiogenesis) triggers a signal cascade that suppresses migration and proliferation of endothelial cells and decreases vascular permeability. Clinically, monthly or bimonthly intravitreal injection of large protein anti‐VEGF drugs, such as bevacizumab, is a major therapeutic strategy for CNV.^[^ ^113^ ^]^ However, anti‐VEGF strategies afford a transient therapeutic outcome, owing to their rapid degradation and poor targeting in dysfunctional existing vessels. These drawbacks necessitate repeated administration to maintain effectiveness and subsequently cause potential infection and tissue injury.^[^ ^7^ ^]^ In addition, ocular nanomedicine‐assisted procedures have been successfully engineered with inherent antiangiogenic properties derived from a natural product, administered via eye drops or conjunctival sac instillation to achieve minimally invasive intraocular delivery, or via targeted and sustained release of anti‐VEGF peptide/antibody and siRNA‐oriented intracellular therapeutic agents at therapeutically relevant concentrations to minimize dosage intervals.^[^ ^80^, ^82^, ^114^ ^]^ Reportedly, externally triggered targeted delivery allows ocular drug delivery with high temporal and spatial resolution. Among external stimulators, light is particularly attractive as an energy source for retinal targeting, as the eye is naturally used to admit light.^[^ ^1^ ^]^ Wang et al. designed smart photo‐targeted nanoparticles by self‐assembling a chemically modified PEG‐PLA block copolymer, further modified with a targeting moiety, such as a cell‐penetrating peptide for high cellular uptake. After intravenous administration, the nanoparticles are converted to a tissue‐targeting state depending on the irradiation in the eye. This strategy allows the noninvasive treatment of CNV by allowing target drug accumulation in the diseased ocular areas, simultaneously minimizing drug deposition at off‐target sites in healthy ocular/systemic sites.^[^ ^36b^ ^]^ Additionally, oxidative stress and inflammation play a crucial pathogenic role in angiogenesis during AMD pathogenesis. Mitra et al. designed glycol chitosan‐coated antioxidant ceria nanoparticles, which were autoregenerative and more active in blocking laser‐induced CNV models. On downregulating ROS‐activated pro‐angiogenic VEGF expression, the nanoparticles significantly rescued the cumulative oxidative damage and recruited endothelial precursor cells without affording any toxicity (**Figure** **15**).^[^ ^99b^ ^]^ Overall, the ocular nanomedicine‐assisted strategy provides an alternative intervention to optimally inhibit ocular neovascularization without damaging healthy tissues in a non‐invasive or minimally invasive manner while reducing the repeated administration to establish therapeutic control.

### 5.5 Ocular Tissue Engineering

The structure and function of injured cells, tissues, and organs after experiencing trauma, infection, or inflammation can be damaged if healing remains unsatisfactory. For example, retinal degeneration is recognized as one predominant cause underlying irreversible visual impairment and even blindness worldwide, and the death of RPE and/or photoreceptors has been considered common pathogenesis.^[^ ^115^ ^]^ Hence, RPE/photoreceptor‐based transplantation into diseased sites is necessary to generate monolayer cells and recover the RPE‐photoreceptor interface. Over the past two decades, ocular tissue engineering, including corneal, conjunctival, and retinal regeneration, has been shown to be feasible by transplanting cell/tissue‐derived implants. However, these are limited in terms of specific responses to biological cues and superior reconstruction of normal functions.^[^ ^116^ ^]^ However, ocular nanomedicine‐based strategies could introduce and guide ocular tissue regeneration by stimulating the signaling pathway by communicating with surroundings or serving as a sensor to monitor/trace transplanted cell replacement and tissue repair.^[^ ^117^ ^]^ As an emerging nanomaterial, tetrahedral framework nucleic acids (tFNAs) accelerated the re‐epithelialization by promoting the migration and proliferation of human corneal epithelial cells. Moreover, tFNAs showed high efficacy in recovering corneal transparency and corneal wound healing in an established corneal alkali burn animal model.^[^ ^118^ ^]^ In retinal tissue engineering, a micropatterned PLGA nanosheet with perfect flexibility has been adopted to achieve a good microenvironment for the stable attachment of a transplanted RPE monolayer. By further embedding with magnetic nanoparticles, the nanosheet promoted malleability, optical visualization, and cell morphogenesis. After subretinal injection into the swine eye, this nanosheet exhibited stable attachment to the macula, and the implanted RPE monolayer could differentiate into a cobblestone‐like structure.^[^ ^119^ ^]^ Thus, nanomedicine‐aided ocular regenerative medicine is expected to establish an innovative therapeutic strategy by guiding the uninjurious cellular organization.

**Figure 16:**
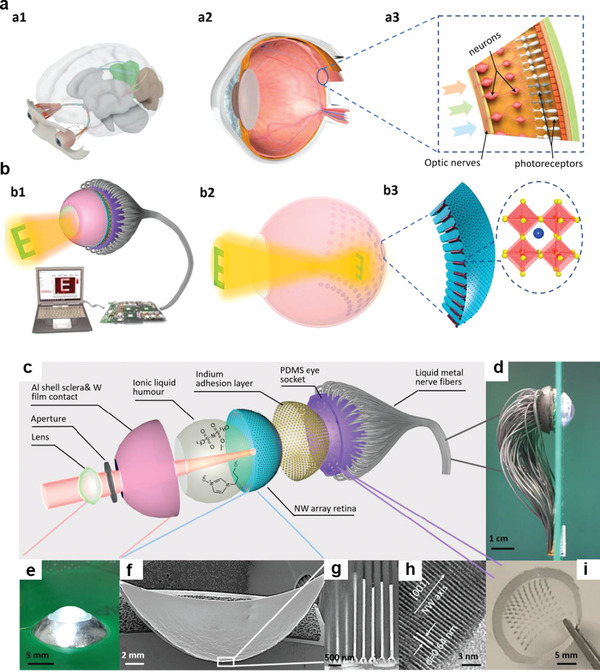
Biomimetic electrochemical eye (EC‐EYE) with hemispherical perovskite nanowire array retina. a,b) The overall comparison of human and EC‐EYE imaging systems. Schematic illustration of human visual system (a1), human eye (a2), and human retina (a3). Schematic illustration of EC‐EYE imaging system (b1), working mechanism of EC‐EYE (b2), and nanowire in hemispherical porous‐alumina‐membrane (PAM) template and molecular structure (b3). c–i) Detailed structure of EC‐EYE: c) Layered structure, d) side‐view, and e) top‐view of a completed EC‐EYE. f) Low‐resolution cross‐sectional SEM image of hemispherical PAM/nanowire. g) Cross‐sectional SEM images of nanowire in PAM. h) Representative high‐resolution transmission electron microscope (TEM) image of nanowire. i) Photo of polydimethylsiloxane socket assisting the alignment of liquid metal wires. Reproduced with permission.^[^ ^120a^ ^]^ Copyright 2020, Nature Publishing Group.

Human eyes have exceptional image sensing functions, such as high sensitivity and resolution with low aberration and a spectacularly wide field of view.^[^ ^1^ ^]^ During the process, the retina efficiently detects light stimuli and pre‐processes image information in parallel, prior to the brain performing more complex actions.^[^ ^1^ ^]^ Biomimetic eyes with similar superior features are extremely desirable for different technological applications. In recent years, digital vision systems based on charge‐coupled device cameras or conventional, complementary metal‐oxide‐semiconductor imagers have been explored to fulfill computer vision by interface‐interfaced digital processing units on coarsely parallel or serial structures.^[^ ^120^ ^]^ Recently, a unique biomimetic electrochemical eye (EC‐EYE) using a hemispherical retina has been constructed based on a high‐density array of perovskite nanowires to mimic photoreceptors on an actual retina. A front‐side common contact with nanowires is the ionic liquid electrolyte, and the back‐side contact with the nanowire photosensors is a liquid metal wire to mimic human nerve fibers behind the retina. The artificial visual system possesses an image sensing function and structural similarity to the human eye, with the capacity to obtain a high imaging resolution when individual nanowires are electrically addressed (**Figure** **16**).^[^ ^120a^ ^]^ However, these digital, artificial vision systems are expensive and markedly large for practical applications, as well as consume considerable power. Thus, a new generation of imaging and photosensing neuromorphic vision sensors, based on novel nanomedicine with high detectivity, ultrahigh responsivity, and signal‐to‐noise ratio, could overcome these disadvantages.

### 5.6 Ocular Cancer Therapy

**Figure 17:**
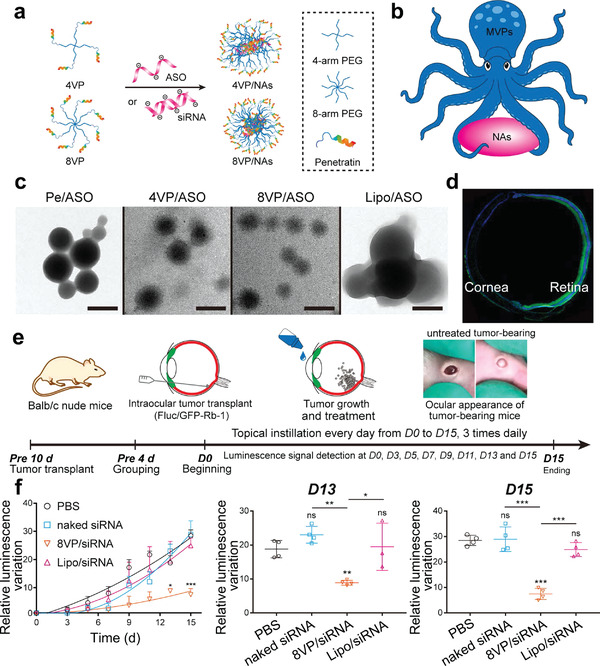
Noninvasive delivery of nucleic acids (NAs) upon octopus‐like flexible vector for retinoblastoma treatment. a) Construction of polyplexes by a simple mixing of multivalent penetratin (MVP) and NAs, for example, siRNA or antisense oligonucleotides (ASO). b) Several cationic penetratin tentacles in the polyplexes can freely bind with anionic NAs, whereas others regulated the noninvasive intraocular delivery, similar to an octopus carrying NAs and moving forward. c) TEM images showed the morphology of polyplexes. Scale bar, 200 nm. d) Intraocular distribution of ASO delivered by 8‐valent penetratin (8VP) polyplexes in the whole mice eyes including retinas and corneas. e) Establishment and therapy of retinoblastoma‐bearing mice model. f) Semiquantitative inhibition effects of different polyplexes on bioluminescence expression of the tumor. Reproduced with permission.^[^ ^123^ ^]^ Copyright 2019, American Chemical Society.

Retinoblastoma is an aggressive and malignant intraocular tumor in infancy and childhood, whereas uveal melanoma (UM) typically occurs in the choroid, iris, and ciliary body, the most common intraocular cancer in adults.^[^ ^121^ ^]^ The considerably high mortality rate of retinoblastoma and UM warrant the urgent development of efficacious therapeutics. Currently available medical interventions primarily include enucleation (eye removal), external beam/episcleral plaque radiotherapy, chemoreduction, and chemotherapy.^[^ ^121^ ^]^ Enucleation is the only suitable approach for advanced retinoblastoma, but surgical lesions result in facial deformities. Radiotherapy can induce various side effects, such as dry eye, which requires permanent eye lubrication. Although chemoreduction overcomes several shortcomings associated with other procedures, it can induce severe complications, including temporary pancytopenia, renal toxicity, and secondary cancer. Currently, chemotherapy has been widely utilized to treat ocular cancer, using individual or synergetic administration of anticancer drugs, such as topotecan and vincristine. However, these chemotherapeutics are highly cytotoxic and can cause adverse effects and secondary complications. Moreover, the distinct physiological and anatomical arrangement of the eye presents an enormous barrier for the targeted transport of drugs to cancer tissues. The recent advancement of a single combinatorial nanotechnology to target ocular cancer cells has afforded an unprecedented possibility of reducing off‐target effects and boosting synergistic anti‐tumor efficacy. Accordingly, various biomaterial‐based nanoplatforms composed of nanoceria, MSNs, AuNPs, and functionalized micelles have been extensively exploited in ocular cancer. These materials exert effects by delivering chemotherapy drugs and/or targeted tumor molecules (e.g., microRNAs, oxygen) in response to the extracellular pH to enhance chemotherapeutic activity or excited by two photons to improve photodynamic therapy.^[^ ^122^ ^]^ An octopus‐like 8‐valent penetratin (8VP < 100 nm) has been designed with extremely flexible cationic penetratin tentacles and branched spatial structure to effectively deliver siRNA or ASO into retinoblastoma‐bearing mice by promoting their stability. This non‐viral vector nanostructure presented overwhelming advantages when compared with commercial transfection reagents in terms of both efficiency and safety by significantly improving cancer cellular internalization (approaching 100%) and transfection rate (over 75%), thus enriching the non‐invasive gene delivery technologies for retinoblastoma treatment (**Figure** **17**).^[^ ^123^ ^]^ One drawback of ocular radiotherapy is the difficulty in selectively killing retinoblastoma cells during laser irradiation, which may cause injury to healthy ocular tissues, especially the cornea. Accordingly, biocompatible magnesium oxide nanoparticles with desirable thermal conductivities were obtained by computation. The laser beam was emitted after nanoparticle injection along the pupillary axis, and the amount of generated heat was directly proportional to the absorption coefficient in each eye segment. It can be postulated that low‐thermal‐conductive nanoparticles will accumulate around the tumor mass to effectively avoid the protrusion of the lethal zone from the tumor lesion, thus protecting healthy eye tissues from potential hazards of thermal damage.^[^ ^124^ ^]^ To date, versatile applications of ocular nanomedicine, including synergetic chemotherapeutic, photodynamic therapy, and selective nanothermal therapy for ocular tumors, especially retinoblastoma and UM, remains in the infant stage; therefore, additional advances in nanotechnology are urgently required to provide less toxic, minimally invasive, and highly effective management.

### 5.7 Refractive Error Correction

**Figure 18:**
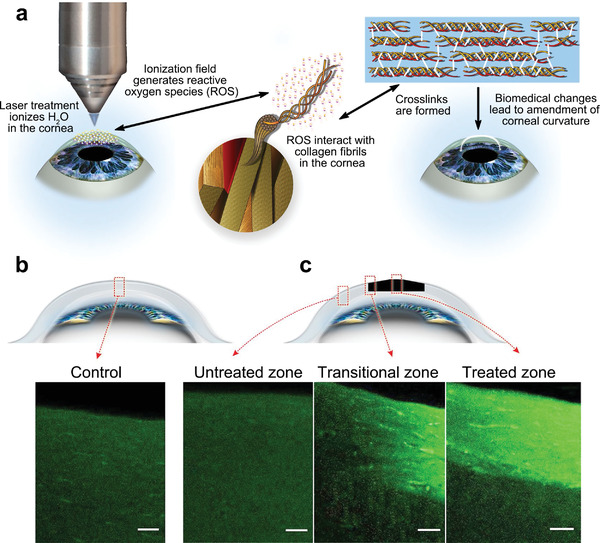
Noninvasive permanent refractive error correction by femtosecond laser‐triggered reactive oxygen species (ROS) generation. a) Schematic illustration of laser‐assisted therapeutic process, which is associated with the low‐density plasma production, ROS, and cross‐link formation. b,c) Differences in structure between control and laser‐treated pig eyes. Two‐photon fluorescence (TPF) images of the cross‐sections of control (b) and laser‐treated (c) pig eyes. There are three zones imaged in the laser‐treated eye: an untreated zone (left), a transitional zone (middle), and the central zone (right). Crosslink density in control eyes is similar to that in untreated zone of the laser‐irradiated specimen. Scale bars, 60 µm. Reproduced with permission.^[^ ^129^ ^]^ Copyright 2018, Nature Publishing Group.

Refractive error of the cornea, particularly myopia (i.e., short‐sightedness), is a major cause of recoverable/reversible visual impairment worldwide, and a sharp increase in myopia‐related morbidity is inevitable in modern society.^[^ ^125^ ^]^ Most patients with refractive error wear spectacles to afford refractive error correction; however, wearing spectacles could cause great inconvenience in daily life.^[^ ^126^ ^]^ Recently, emerging refractive surgeries, especially laser‐assisted in situ keratomileusis (LASIK) surgery), are considered an attractive choice for permanent vision correction. However, a follow‐up investigation of long‐term safety post‐surgery is insufficient, and invasive surgery may cause thin ectatic corneas and corneal flap bonding post‐LASIK surgery, leading to inefficient refractive error correction.^[^ ^126^ ^]^ Accordingly, two‐photon collagen cross‐linking (2P‐CXL) of intact corneal tissue has been designed using riboflavin and femtosecond laser irradiation. The results revealed that disturbing surrounding non‐irradiated regions exerted no effect on the visualization of the cross‐linked pattern, and the 2P‐CXL‐induced stiffening was similar to that observed in the conventional one‐photon CXL. The 2P‐CXL technique allows selective stiffening of corneal tissue in situ at high spatial resolution and permits 3D control of cross‐linking by nonlinear excitation, which can be readily applied to assist LASIK surgery and selectively modulate corneal curvature for vision error treatment.^[^ ^127^ ^]^ As an alternative to irreversible LASIK surgery, the application of the Abbe number, high visible light transmittance, and refractive index in contact lenses affords great potential for refractive error correction. Shaker et al. used titanium dioxide nanoparticles (TiO_2_) as a suitable candidate to maintain high transparency and increase the refractive index of poly(methyl methacrylate)‐TiO_2_ contact lenses. Contact lenses with higher doping content contributed to the best vision correction and reduced the generated spherical and chromatic aberrations in the aberrant eye.^[^ ^128^ ^]^ Additionally, Wang et al. proposed another non‐invasive approach for permanent vision correction based on the femtosecond laser. This strategy produces low‐density plasma and ROS within collagenous tissues to assist the oxidation of surrounding proteins and cross‐link formation, ultimately initiating the alteration of refractive power in the eyes (**Figure** **18**).^[^ ^129^ ^]^ This pioneering strategy inspired scientists to propose similar feasible ROS‐producing nanomedicines to improve therapeutic outcomes and relieve potential side effects, ultimately favoring future clinical translation for refractive error correction.

### 5.8 Ocular Imaging

**Figure 19:**
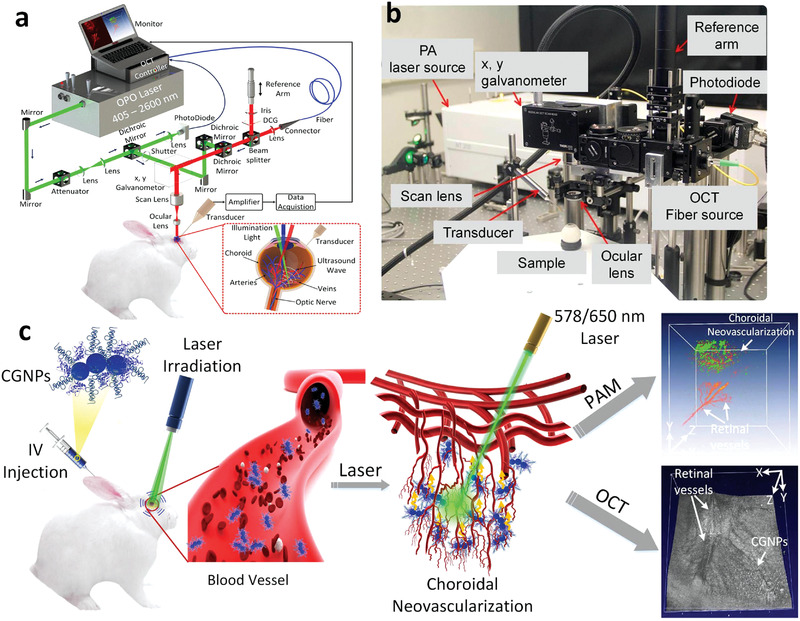
Chain‐like gold nanoparticle (CGNP) clusters for multimodal photoacoustic microscopy (PAM) and OCT enhanced molecular imaging. Experimental setup of PAM and OCT equipment. a) Schematic illustration of the imaging system. b) Physical setup. In PAM mode, nanosecond excitation laser (450–710 nm) delivered and focused onto the retina. To enable multimodal imaging, the excitation laser beam utilized to induce photoacoustic signal was coaxially aligned with OCT multispectral luminescence with a center wavelength at 805 and 905 nm. The generated acoustic signal was detected by a needle‐shaped hydrophone ultrasonic transducer, and the recorded data was used to reconstruct PAM images. A spectrometer was used to detect the reflected OCT light interfered with the reference light and the interference intensity spectra. Retina was scanned by a galvanometer. c) Illustration of in vivo multimodal imaging after intravenous injection of CGNP clusters‐RGD into the rabbit model. Photoacoustic signals from rabbit retina was produced using nanosecond pulsed laser illumination at 578 or 650 nm. Reproduced under the terms of the CC‐BY 4.0 license.^[^ ^132^ ^]^ Copyright 2020, The Authors. Published by Nature Publishing Group.

Ocular imaging is essential for clinical diagnosis, therapeutics, and real‐time monitoring of ocular disorders at an early stage. For instance, given the rapid progression of intraocular tumors, early imaging diagnosis is necessary to prevent vision loss and even death.^[^ ^130^ ^]^ However, most available therapeutics lack the capability of imaging and diagnosis before the onset of ocular disorders and precise monitoring.^[^ ^8^ ^]^ In ocular disease, nanomedicine‐based strategies subjected to a strict screening could be selectively employed to visualize ocular tissues and accurately and quantitatively examine altered levels of small molecules, proteins, or nucleic acids, ultimately determining the physiological state of ocular tissues.^[^ ^131^ ^]^ Considering that colloidal gold nanoparticles (GNPs) serve as promising contrast agents in photoacoustic imaging, Nguyen et al. designed chain‐like GNPs (CGNP) clusters‐arginine‐glycine‐aspartic acid (RGD) for enhanced molecular imaging by conjugating RGD peptides with ultrapure CGNP clusters, exhibiting a redshift peak wavelength at 650 nm. The synthesized nanoparticles exhibited excellent photostability and biocompatibility and could disassemble to facilitate elimination from the body. Importantly, intravenous injection of CGNP clusters‐RGD via the marginal ear vein bound to CNV, causing an increase of up to 17‐fold in the photoacoustic microscopy signal and a 176% increase in optical coherence tomography signal, which is beneficial for visualizing newly developed blood vessels in the subretinal space (**Figure** **19**).^[^ ^132^ ^]^ Additionally, smart wearable platforms, such as contact lenses combined with functionalized nanomaterials, help either sensitively identify the altered constituent (e.g., cortisol, Na ions, and IOP) or detect microorganisms such as *Staphylococcus aureus*, which play a positive role in early diagnosis and prophylactic therapy of various ocular diseases, including glaucoma, dry eye, and edema.^[^ ^133^ ^]^ Recently, a breakthrough in fabrication methodologies and synthetic chemistry of ocular nanomedicine has facilitated theranostics by integrating imaging diagnosis and therapy, which can help identify the specific period most susceptible to intervention for a given disease.^[^ ^134^ ^]^ The introduced dual‐functional SiNPs‐RGD is a class of high‐quality theranostic probes for synergetic imaging and treatment of ocular neovascularization by marking angiogenic blood vessels while effectively suppressing corneal and retinal neovascularization with negligible toxicity.^[^ ^135^ ^]^ However, efforts to improve theranostic nanomaterials should focus on enhanced imaging modalities and more satisfactory diagnostic and prognostic effects.

## 6 Conclusion and Outlook

The intrinsic limitations of conventional management of ocular diseases have propagated the growth of ocular nanomedicine. Herein, we overview the state‐of‐the‐art ocular nanomedicine, predominantly reported in the last 5 years, and provide an in‐depth analysis of how fundamental physicochemical characteristics of nanomaterials affect their effectiveness after interacting with the extra/intraocular milieu. Further discussion of functionalized ocular nanomedicine by engineering suitable entities has also been conducted. Additionally, diverse applications of ocular nanomedicine to combat a wide range of ocular pathogenesis have been comprehensively presented. Typically, the advantages of nanomedicine used in ophthalmology include: 1) prophylactic and antidotic effects by exerting inherent therapeutic properties, 2) possible permeation across complex ocular barriers, 3) targeted and controllable cargo release by avoiding rapid inactivation and burst effects, 4) on‐demand and co‐delivery of various therapeutic cargos with differentiated physicochemical properties, and 5) synergistic effects by integrating imaging with therapeutics. Although considerable breakthroughs have been achieved, several unresolved scientific issues and corresponding perspectives encountered in the design and clinical translation of ocular nanomedicine require elucidation.

**Figure 20:**
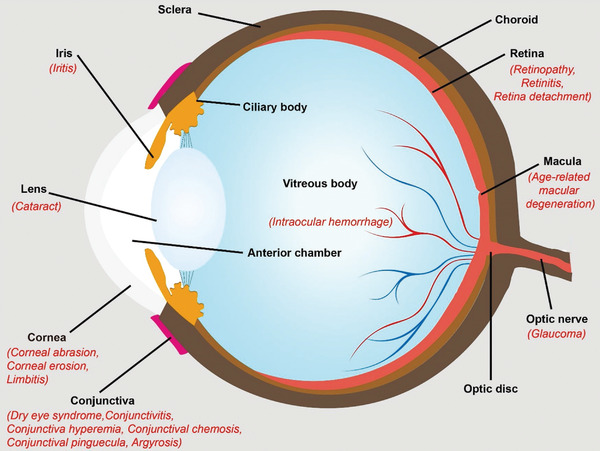
Nanomaterial exposure induced typical eye‐related disorders. Once ocular tissues contact with nanomaterials, potential toxic effects caused by nanomaterials could occur on ocular surface (e.g., cornea), within the eye (e.g., lens and iris) or inner surface of retina, macula optic, and nerve. Reproduced under the terms of the Creative Commons CC‐BY license.^[^ ^56^ ^]^ Copyright 2019, The Authors. Published by Wiley‐VCH.

1) Current trends in ocular nanomedicine have gradually shifted from in vitro evaluations to preliminary in vivo assays in different animal models. However, the anatomical structure/geometry and composition of ocular tissues are distinct among distinct species, particularly among animal models and humans. Thus, in vivo animal models of eye‐related diseases have a roughly predictive role in humans. For instance, the distinctive space and dimension discrepancy of the vitreous body and the proximity of the vitreous body to the retina is known to exist between rodents and humans, making it difficult to translate preclinical outcomes to humans. Moreover, the thicker retina and smaller vitreous volume in mice and rats than in humans make it difficult to precisely evaluate the actual intraocular drug transport and distribution of ocular nanomedicine. As a commonly used animal model of eye disease, rabbits are comparable in size to the human eye, with different physiological behaviors, such as higher mucus production, lower tear production, and higher surface sensitivity. It is also worth noting that simply converting the equivalent dose of nanoparticles from other species does not ensure safety in humans, given the above differences between animals and humans.^[^ ^136^ ^]^ Therefore, considerable attention should be paid to higher mammals for preclinical safety and efficacy studies of ocular nanomedicine, especially in non‐human primates whose eyes are similar to the human eye, as well as to reproduce several clinical symptoms consistent with human patients.
2) Although numerous cellular studies and animal investigations have been performed to determine therapeutic outcomes of designed ocular nanomedicine, it is still challenging to completely clarify the underlying mechanisms of nanomedicine on ocular cells/tissues following biomedical analysis, that is, only therapeutic results, rather than the processes, are established. For instance, regardless of the existing evidence supporting the ultimate therapeutic outcomes of nanomaterials, it can be challenging to quantify their reaction kinetics in vivo. These “black boxes” obviously exist in almost all therapeutic processes associated with nanomedicine used for various biomedical applications. Multifunctional optical probes have been developed to evaluate the distribution and therapeutic concentration of specific biochemical ingredients in the biological milieu.^[^ ^137^ ^]^ These multifunctional diagnostic/therapeutic agents may be beneficial for the precise characterization of therapeutic processes in vivo, consequently clarifying the relationship between ocular nanomedicine and in vivo behavior.
3) The human eye is regarded as one of the most exquisite organs of the body and serves as a perfect platform for nanoparticle delivery, capable of bypassing systemic circulation. Different routes of administration influence nanoparticle biodistribution. Current methods for nanoparticle delivery into the anterior eye segment mainly focus on eye drops; however, frequent blinking and tears limit nanoparticle retention on the ocular surface, leading to poor bioavailability.^[^ ^138^ ^]^ Subconjunctival administration serves as a less invasive option to overcome blood‐ocular barriers and improve drug bioavailability in specific ocular sites.^[^ ^20^ ^]^ Subconjunctival implantation has been well‐examined for periocular implants, but this approach may require a second surgery. As chronic retinal disorders require long‐term intervention, subretinal injection provides a relatively high nanoparticle bioavailability; however, it is highly invasive and may be accompanied by complications such as intraocular bleeding.^[^ ^139^ ^]^ Although the intravitreal procedure avoids anterior eye barriers, the complex inner limiting membrane and vitreoretinal interface may prevent nanoparticle access to the retina. Retinal permeation of intravitreal nanocarriers is limited and probably misleading, given inter‐species differences in retinal structures between animals and humans. In addition, Abrishami et al. recently designed a gelling hypotonic formulation for extended ocular delivery by forming a highly clear and uniform thin layer. Compared with commercial eye drops and conventional thermosensitive gelling formulations, the hypotonic gel layer resisted clearance from blinking and conformed to the ocular surface, thereby enhancing intraocular drug absorption and prolonging ocular surface contact.^[^ ^140^ ^]^ Based on these findings, further development of penetrable nanomaterials that can permeate into intraocular tissues upon direct eye drop could afford a more effective, non‐invasive, and long‐lasting alternative to conventional therapies. In contrast, given the minimum invasiveness and driving capability of a magnetic field, ocular delivery with the aid of a microrobot confers an inherent advantage for targeted ocular treatment. More importantly, Kim et al. developed a bilayer hydrogel microrobot capable of retrieving magnetic nanoparticles (MNPs) using a magnetic field after drug targeted delivery. This procedure effectively avoids the side effects of MNP retention in conventional microrobots. The sheet‐type microrobot with retrieval and recycling ability demonstrates superior potential as a therapeutic agent against intraocular diseases and affords a new ocular delivery paradigm to develop recycled nanomaterials for ocular disease treatment.^[^ ^141^ ^]^
4) As most ocular nanomedicines are unique and under development, a continuous investigation of therapeutic safety and effectiveness is required prior to utilization in clinical practice. Biosafety issues, including genotoxicity, neurotoxicity, and reproductive toxicity of ocular nanomedicine, especially inorganic nanoparticles, remain a prerequisite and top priority.^[^ ^65^ ^]^ In vivo biocompatibility concerns of elaborately constructed nanomaterials, such as unsterile contaminants in contact with various ocular structures, have been comprehensively reviewed by the Zhu group, demonstrating that the biological compatibility of ocular nanomedicine is closely associated with the synthesized structure/morphology, particle sizes, and dosing parameters (**Figure** **20**).^[^ ^56^ ^]^
5) The last and most critical concern associated with ocular nanomedicine is the future clinical translation and successful commercialization. Although numerous fundamental investigations and preclinical evaluations have been reported, to the best of our knowledge, only several types of nanosystems have been approved for clinical trials to combat ocular diseases, including emerging exosome‐based therapeutics for macular edema (Table 2).^[^ ^7^ ^]^ The current large‐scale synthesis of nanomedicines used in biomedical fields is a common issue considering their complex, diverse nanostructures and surface modification, which may be addressed to a certain extent by simplifying the corresponding construction without compromising bioactivity.

**Figure 21:**
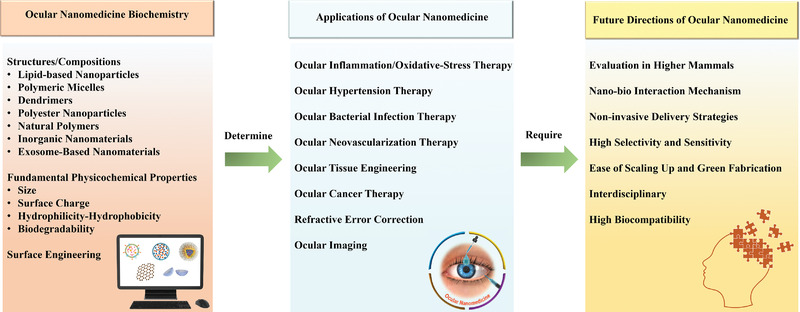
Summary of ocular nanomedicine. The underlying principles of ocular nanomedicine biochemistry play an essential role in determining their subsequent ophthalmologic applications. However, to fulfill specific theranostic goals, some critical challenges remain to be addressed, and corresponding future directions on the developments of ocular nanomedicine need to be clarified.

These challenges and corresponding directions in the progression of both conventional and emerging ocular nanomedicine prompt us to reconsider aspects that should be considered to accelerate clinical translation (**Figure** **21**). Despite continued advances in basic and clinical ophthalmology research and nanomedicine to meet individual patient requirements, it is believed that there remains “plenty of room at the bottom” for developing a satisfactory strategy with broad impact in the future.

## Conflict of Interest

The authors declare no conflict of interest.

## References

[1] Y. Ma , J. Bao , Y. Zhang , Z. Li , X. Zhou , C. Wan , L. Huang , Y. Zhao , G. Han , T. Xue , Cell 2019, 177, 243. 30827682 10.1016/j.cell.2019.01.038

[2] a) X. Wang , N. Lou , A. Eberhardt , Y. Yang , P. Kusk , Q. Xu , B. Förstera , S. Peng , M. Shi , A. Ladrón‐de‐Guevara , C. Delle , B. Sigurdsson , A. L. R. Xavier , A. Ertürk , R. T. Libby , L. Chen , A. S. Thrane , M. Nedergaard , Sci. Transl. Med. 2020, 12, eaaw3210; 32213628 10.1126/scitranslmed.aaw3210 PMC7356596

[3] M. J. Burton , J. Ramke , A. P. Marques , R. R. A. Bourne , N. Congdon , I. Jones , B. A. M. Ah Tong , S. Arunga , D. Bachani , C. Bascaran , A. Bastawrous , K. Blanchet , T. Braithwaite , J. C. Buchan , J. Cairns , A. Cama , M. Chagunda , C. Chuluunkhuu , A. Cooper , J. Crofts‐Lawrence , W. H. Dean , A. K. Denniston , J. R. Ehrlich , P. M. Emerson , J. R. Evans , K. D. Frick , D. S. Friedman , J. M. Furtado , M. M. Gichangi , S. Gichuhi , et al., Lancet Glob. Health. 2021, 9, e489.33607016

[4] N. Ren , R. Sun , K. Xia , Q. Zhang , W. Li , F. Wang , X. Zhang , Z. Ge , L. Wang , C. Fan , Y. Zhu , ACS Appl. Mater. Interfaces 2019, 11, 26704. 31264833 10.1021/acsami.9b08652

[5] H. Zierden , A. Josyula , R. Shapiro , H. Hsueh , J. Hanes , L. Ensign , Trends Mol. Med. 2021, 27, P436. 10.1016/j.molmed.2020.12.001 PMC8087626 33414070

[6] X. Liu , A. Guo , Y. Tu , W. Li , L. Li , W. Liu , Y. Ju , Y. Zhou , A. Sang , M. Zhu , Cell Death Dis. 2020, 11, 1016. 33247124 10.1038/s41419-020-03222-1 PMC7695853

[7] A. Mandal , D. Pal , V. Agrahari , H. M. Trinh , M. Joseph , A. K. Mitra , Adv. Drug Delivery Rev. 2018, 126, 67. 10.1016/j.addr.2018.01.008 PMC5995646 29339145

[8] J. Li , S. Yang , Z. Liu , G. Wang , P. He , W. Wei , M. Yang , Y. Deng , P. Gu , X. Xie , Z. Kang , G. Ding , H. Zhou , X. Fan , Adv. Mater. 2021, 33, 2005096. 10.1002/adma.202005096 33244820

[9] J. He , Q. Wei , S. Wang , S. Hua , M. Zhou , Biomaterials 2021, 271, 120734. 33647873 10.1016/j.biomaterials.2021.120734

[10] a) O. Kari , S. Tavakoli , P. Parkkila , S. Baan , R. Savolainen , T. Ruoslahti , N. Johansson , J. Ndika , H. Alenius , T. Viitala , A. Urtti , T. Lajunen , Pharmaceutics 2020, 12, 763; 10.3390/pharmaceutics12080763 PMC7465487 32806740

[11] B. Yang , Y. Chen , J. Shi , Adv. Healthcare Mater. 2018, 7, 1800268.

[12] M. P. Stewart , A. Sharei , X. Ding , G. Sahay , Nature 2016, 538, 183. 27734871 10.1038/nature19764

[13] Y. Xu , R. Xu , Z. Wang , Y. Zhou , Q. Shen , W. Ji , D. Dang , L. Meng , B. Tang , Chem. Soc. Rev. 2021, 50, 667. 33313632 10.1039/d0cs00676a

[14] A. Shoval , A. Markus , Z. Zhou , X. Liu , R. Cazelles , I. Willner , Y. Mandel , Small 2019, 15, 1902776. 10.1002/smll.201902776 31402576

[15] C. Zhan , C. M. Santamaria , W. Wang , J. B. McAlvin , D. S. Kohane , Biomaterials 2018, 181, 372. 30099260 10.1016/j.biomaterials.2018.07.054 PMC7482074

[16] M. Chang , Y. Kuo , K. Hung , C. Peng , K. Chen , L. Yeh , Biomed. Mater. 2020, 15, 055022. 32434164 10.1088/1748-605X/ab9510

[17] S. Tavakoli , K. Peynshaert , T. Lajunen , J. Devoldere , E. Del Amo , M. Ruponen , S. De Smedt , K. Remaut , A. Urtti , J. Controlled Release 2020, 328, 952. 10.1016/j.jconrel.2020.10.028 33091527

[18] a) J. Paek , S. Park , Q. Lu , K. Park , M. Cho , J. Oh , K. Kwon , Y. Yi , J. Song , H. Edelstein , J. Ishibashi , W. Yang , J. Myerson , R. Kiseleva , P. Aprelev , E. Hood , D. Stambolian , P. Seale , V. Muzykantov , D. Huh , ACS Nano 2019, 13, 7627; 31194909 10.1021/acsnano.9b00686

[19] C. W. Wong , E. Wong , J. M. Metselaar , G. Storm , T. T. Wong , Drug Delivery Transl. Res. 2021. 10.1007/s13346-021-00912-x 33569720

[20] S. Y. Chaw , W. Novera , A. M. Chacko , T. T. L. Wong , S. Venkatraman , J. Controlled Release 2021, 329, 162. 10.1016/j.jconrel.2020.11.053 33271203

[21] K. Sun , K. Hu , Drug Des. Dev. Ther. 2021, 15, 141. 10.2147/DDDT.S287721 PMC7811375 33469266

[22] a) P. S. Apaolaza , A. Del Pozo‐Rodriguez , M. A. Solinis , J. M. Rodriguez , U. Friedrich , J. Torrecilla , B. H. Weber , A. Rodriguez‐Gascon , Biomaterials 2016, 90, 40; 26986855 10.1016/j.biomaterials.2016.03.004

[23] a) E. Aytekin , N. Öztürk , İ. Vural , H. K. Polat , H. B. Çakmak , S. Çalış , S. B. Pehlivan , J. Controlled Release 2020, 324, 238; 10.1016/j.jconrel.2020.05.017 32413453

[24] T. Morita , S. Mukaide , Z. Chen , K. Higashi , H. Imamura , K. Moribe , T. Sumi , Nano Lett. 2021, 21, 1303. 33480258 10.1021/acs.nanolett.0c03978

[25] a) A. Mandal , R. Bisht , I. D. Rupenthal , A. K. Mitra , J. Controlled Release 2017, 248, 96; 10.1016/j.jconrel.2017.01.012 PMC5319397 28087407

[26] S. Schnichels , J. Hurst , J. W. de Vries , S. Ullah , K. Frößl , A. Gruszka , M. Löscher , K. U. Bartz‐Schmidt , M. S. Spitzer , A. Herrmann , ACS Appl. Mater. Interfaces 2021, 13, 9445. 33528240 10.1021/acsami.0c18626

[27] B. Begines , T. Ortiz , M. Pérez‐Aranda , G. Martínez , M. Merinero , F. Argüelles‐Arias , A. Alcudia , Nanomaterials 2020, 10, 1403. 10.3390/nano10071403 PMC7408012 32707641

[28] Y. H. Weng , X. W. Ma , J. Che , C. Li , J. Liu , S. Z. Chen , Y. Q. Wang , Y. L. Gan , H. Chen , Z. B. Hu , K. H. Nan , X. J. Liang , Adv. Sci. 2018, 5, 1700455. 10.1002/advs.201700455 PMC5770669 29375972

[29] A. Mandal , K. Cholkar , V. Khurana , A. Shah , V. Agrahari , R. Bisht , D. Pal , A. K. Mitra , Mol. Pharmaceutics 2017, 14, 2056. 10.1021/acs.molpharmaceut.7b00128 28471177

[30] C. Qin , S. Wen , S. Zhu , D. Liu , S. Chen , J. Qie , H. Chen , Q. Lin , J. Ocul. Pharmacol. Ther. 2020, 36, 715. 33121321 10.1089/jop.2020.0078

[31] I. Heredero‐Bermejo , T. Martín‐Pérez , J. Copa‐Patiño , R. Gómez , F. de la Mata , J. Soliveri , J. Pérez‐Serrano , Pharmaceutics 2020, 12, 565. 10.3390/pharmaceutics12060565 PMC7356815 32570829

[32] U. Soiberman , S. P. Kambhampati , T. Wu , M. K. Mishra , Y. Oh , R. Sharma , J. Wang , A. E. Al Towerki , S. Yiu , W. J. Stark , R. M. Kannan , Biomaterials 2017, 125, 38. 28226245 10.1016/j.biomaterials.2017.02.016 PMC5870122

[33] X. Zhou , J. Lv , G. Li , T. Qian , H. Jiang , J. Xu , Y. Cheng , J. Hong , Biomaterials 2021, 268, 120600. 33360507 10.1016/j.biomaterials.2020.120600

[34] a) X. Yang , L. Wang , L. Li , M. Han , S. Tang , T. Wang , J. Han , X. He , X. He , A. Wang , K. Sun , Drug Delivery 2019, 26, 989; 31571502 10.1080/10717544.2019.1667455 PMC6781193

[35] A. Sharma , R. Sharma , Z. Zhang , K. Liaw , S. P. Kambhampati , J. E. Porterfield , K. C. Lin , L. B. DeRidder , S. Kannan , R. M. Kannan , Sci. Adv. 2020, 6, eaay8514. 32010790 10.1126/sciadv.aay8514 PMC6976300

[36] a) E. Sánchez‐López , M. A. Egea , B. M. Davis , L. Guo , M. Espina , A. M. Silva , A. C. Calpena , E. M. B. Souto , N. Ravindran , M. Ettcheto , A. Camins , M. L. García , M. F. Cordeiro , Small 2018, 14; 10.1002/smll.201701808 29154484

[37] G. Jiang , H. Jia , J. Qiu , Z. Mo , Y. Wen , Y. Zhang , Y. Wen , Q. Xie , J. Ban , Z. Lu , Y. Chen , H. Wu , Q. Ni , F. Chen , J. Lu , Z. Wang , H. Li , J. Chen , Int. J. Nanomed. 2020, 15, 9373. 10.2147/IJN.S272750 PMC7699454 33262593

[38] V. A. N. Huu , J. Luo , J. Zhu , J. Zhu , S. Patel , A. Boone , E. Mahmoud , C. McFearin , J. Olejniczak , C. G. Lux , J. Lux , N. Fomina , M. Huynh , K. Zhang , A. Almutairi , J. Controlled Release 2015, 200, 71. 10.1016/j.jconrel.2015.01.001 PMC4384820 25571784

[39] A. Than , C. Liu , H. Chang , P. K. Duong , C. M. G. Cheung , C. Xu , X. Wang , P. Chen , Nat. Commun. 2018, 9, 4433. 30401883 10.1038/s41467-018-06981-w PMC6219513

[40] J. Van Hoorick , J. Delaey , H. Vercammen , J. Van Erps , H. Thienpont , P. Dubruel , N. Zakaria , C. Koppen , S. Van Vlierberghe , B. Van den Bogerd , Adv. Healthcare Mater. 2020, 9, 2000760. 10.1002/adhm.202000760 32603022

[41] L. Li , C. Lu , L. Wang , M. Chen , J. White , X. Hao , K. M. McLean , H. Chen , T. C. Hughes , ACS Appl. Mater. Interfaces 2018, 10, 13283. 29620862 10.1021/acsami.7b17054

[42] Y. Y. Chun , Z. L. Yap , L. F. Seet , H. H. Chan , L. Z. Toh , S. W. L. Chu , Y. S. Lee , T. T. Wong , T. T. Y. Tan , Sci. Rep. 2021, 11, 1470. 33446775 10.1038/s41598-020-80542-4 PMC7809290

[43] A. Balijepalli , R. Sabatelle , M. Chen , B. Suki , M. Grinstaff , Angew. Chem. Int. Ed. Engl. 2020, 59, 704. 31701611 10.1002/anie.201911720 PMC7754715

[44] B. Li , J. Wang , Q. Gui , H. Yang , Bioact. Mater. 2020, 5, 577. 32405573 10.1016/j.bioactmat.2020.04.013 PMC7210375

[45] C. He , T. Ye , W. Teng , Z. Fang , W. S. Ruan , G. Liu , H. Chen , J. Sun , L. Hui , F. Sheng , D. Pan , C. Yang , Y. Zheng , M. B. Luo , K. Yao , B. Wang , ACS Nano 2019, 13, 1910. 30747513 10.1021/acsnano.8b08151

[46] Q. Jin , W. Zhu , J. Zhu , J. Zhu , J. Shen , Z. Liu , Y. Yang , Q. Chen , Adv. Mater. 2021, 33, 2007557. 10.1002/adma.202007557 33448035

[47] a) F. Yu , M. Zheng , A. Y. Zhang , Z. Han , J. Controlled Release 2019, 315, 40; 10.1016/j.jconrel.2019.10.039 PMC6925533 31669212

[48] a) A. Baker , H. Cui , B. Ballios , S. Ing , P. Yan , J. Wolfer , T. Wright , M. Dang , N. Gan , M. Cooke , A. Ortín‐Martínez , V. Wallace , D. van der Kooy , R. Devenyi , M. Shoichet , Biomaterials 2021, 271, 120750; 33725584 10.1016/j.biomaterials.2021.120750

[49] a) S. Raveendran , H. T. Lim , T. Maekawa , M. Vadakke Matham , D. Sakthi Kumar , Nanoscale 2018, 10, 13959; 29700547 10.1039/c8nr02866d

[50] Y. Pang , C. Wei , R. Li , Y. Wu , W. Liu , F. Wang , X. Zhang , X. Wang , Int. J. Nanomed. 2019, 14, 5125. 10.2147/IJN.S192407 PMC6628948 31371951

[51] F. Sauvage , J. C. Fraire , K. Remaut , J. Sebag , K. Peynshaert , M. Harrington , F. J. Van de Velde , R. Xiong , M. J. Tassignon , T. Brans , K. Braeckmans , S. C. De Smedt , ACS Nano 2019, 13, 8401. 31287662 10.1021/acsnano.9b04050

[52] a) D. Yan , Q. Yao , F. Yu , L. Chen , S. Zhang , H. Sun , J. Lin , Y. Fu , Mater. Sci. Eng. C 2020, 111, 110767; 10.1016/j.msec.2020.110767 32279789

[53] M. Anbukkarasi , P. A. Thomas , J. R. Sheu , P. Geraldine , Biomed. Pharmacother. 2017, 91, 467. 28477463 10.1016/j.biopha.2017.04.079

[54] a) K. Kalishwaralal , E. Banumathi , S. R. Kumar Pandian , V. Deepak , J. Muniyandi , S. H. Eom , S. Gurunathan , Colloids Surf. B 2009, 73, 51; 10.1016/j.colsurfb.2009.04.025 19481908

[55] a) L. Luo , D. Nguyen , J. Lai , Biomaterials 2020, 243, 119961; 32171102 10.1016/j.biomaterials.2020.119961

[56] S. Zhu , L. Gong , Y. Li , H. Xu , Z. Gu , Y. Zhao , Adv. Sci. 2019, 6, 1802289. 10.1002/advs.201802289 PMC6702629 31453052

[57] H. Li , X. Wang , X. Zhao , G. Li , F. Pei , H. Zhang , Y. Tan , F. Chen , Small 2020, 16, 2004677. 10.1002/smll.202004677 32939988

[58] a) H. Bahmani Jalali , M. Mohammadi Aria , U. M. Dikbas , S. Sadeghi , B. Ganesh Kumar , M. Sahin , I. H. Kavakli , C. W. Ow‐Yang , S. Nizamoglu , ACS Nano 2018, 12, 8104; 30020770 10.1021/acsnano.8b02976 PMC6117749

[59] a) C. Choi , M. Choi , S. Liu , M. Kim , O. Park , C. Im , J. Kim , X. Qin , G. Lee , K. Cho , M. Kim , E. Joh , J. Lee , D. Son , S. Kwon , N. Jeon , Y. Song , N. Lu , D. Kim , Nat. Commun. 2017, 8, 1664; 29162854 10.1038/s41467-017-01824-6 PMC5698290

[60] S. Lee , I. Jo , S. Kang , B. Jang , J. Moon , J. B. Park , S. Lee , S. Rho , Y. Kim , B. H. Hong , ACS Nano 2017, 11, 5318. 28199121 10.1021/acsnano.7b00370

[61] J. Y. Kim , J. H. Park , M. Kim , H. Jeong , J. Hong , R. S. Chuck , C. Y. Park , Sci. Rep. 2017, 7, 14566. 29109483 10.1038/s41598-017-15247-2 PMC5674045

[62] J. G. Sun , Q. Jiang , X. P. Zhang , K. Shan , B. H. Liu , C. Zhao , B. Yan , J. Nanomed. 2019, 14, 1489. 10.2147/IJN.S195504 PMC6396882 30880960

[63] W. Qu , B. Meng , Y. Yu , S. Wang , Mater. Sci. Eng. C 2017, 76, 646. 10.1016/j.msec.2017.03.036 28482574

[64] X. Chen , S. Zhu , X. Hu , D. Sun , J. Yang , C. Yang , W. Wu , Y. Li , X. Gu , M. Li , B. Liu , L. Ge , Z. Gu , H. Xu , Nanoscale 2020, 12, 13637. 32567638 10.1039/d0nr03208e

[65] S. F. Hansen , A. Lennquist , Nat. Nanotechnol. 2020, 15, 3. 31925393 10.1038/s41565-019-0613-9

[66] X. Zhang , J. Liu , B. Yu , F. Ma , X. Ren , X. Li , Graefes. Arch. Clin. Exp. Ophthalmol. 2018, 256, 2041. 30167916 10.1007/s00417-018-4097-3

[67] a) B. Mead , S. Tomarev , Stem Cells Transl. Med. 2017, 6, 1273; 28198592 10.1002/sctm.16-0428 PMC5442835

[68] B. Mathew , S. Ravindran , X. Liu , L. Torres , M. Chennakesavalu , C. C. Huang , L. Feng , R. Zelka , J. Lopez , M. Sharma , S. Roth , Biomaterials 2019, 197, 146. 30654160 10.1016/j.biomaterials.2019.01.016 PMC6425741

[69] S. J. Wassmer , L. S. Carvalho , B. Gyorgy , L. H. Vandenberghe , C. A. Maguire , Sci. Rep. 2017, 7, 45329. 28361998 10.1038/srep45329 PMC5374486

[70] B. Mead , S. Tomarev , Prog. Retinal Eye Res. 2020, 79, 100849. 10.1016/j.preteyeres.2020.100849 PMC9460937 32169632

[71] B. Yang , Y. Chen , J. Shi , Adv. Mater. 2019, 31, 1802896. 10.1002/adma.201802896 30126052

[72] R. Robinson , S. R. Viviano , J. M. Criscione , C. A. Williams , L. Jun , J. C. Tsai , E. B. Lavik , ACS Nano 2011, 5, 4392. 21619059 10.1021/nn103146p PMC3136352

[73] S. Patel , J. Kim , M. Herrera , A. Mukherjee , A. V. Kabanov , G. Sahay , Adv. Drug Delivery Rev. 2019, 144, 90. 10.1016/j.addr.2019.08.004 PMC6986687 31419450

[74] S. Taghe , S. Mirzaeei , R. G. Alany , A. Nokhodchi , Biomedicines 2020, 8, 466. 10.3390/biomedicines8110466 PMC7692161 33142768

[75] a) X. Ding , G. Ben‐Shlomo , L. Que , ACS Appl. Mater. Interfaces 2020, 12, 45789; 32960561 10.1021/acsami.0c12667

[76] a) L. Tai , C. Liu , K. Jiang , X. Chen , G. Wei , W. Lu , W. Pan , Nanomedicine 2017, 13, 2091; 28435135 10.1016/j.nano.2017.04.011

[77] a) R. Varela‐Fernández , X. García‐Otero , V. Díaz‐Tomé , U. Regueiro , M. López‐López , M. González‐Barcia , M. I. Lema , F. J. Otero‐Espinar , ACS Appl. Mater. Interfaces 2021, 13, 3559; 33428398 10.1021/acsami.0c18926

[78] H. Han , Y. Gao , M. Chai , X. Zhang , S. Liu , Y. Huang , Q. Jin , A. Grzybowski , J. Ji , K. Yao , J. Controlled Release 2020, 327, 676. 10.1016/j.jconrel.2020.09.014 32920078

[79] a) T. F. Martens , K. Remaut , H. Deschout , J. F. J. Engbersen , W. E. Hennink , M. J. v. Steenbergen , J. Demeester , S. C. D. Smedt , K. Braeckmans , J. Controlled Release 2015, 202, 83; 10.1016/j.jconrel.2015.01.030 25634806

[80] B. Senturk , M. O. Cubuk , M. C. Ozmen , B. Aydin , M. O. Guler , A. B. Tekinay , Biomaterials 2016, 107, 124. 27616429 10.1016/j.biomaterials.2016.08.045

[81] Y. Wang , P. K. Shahi , R. Xie , H. Zhang , A. A. Abdeen , N. Yodsanit , Z. Ma , K. Saha , B. R. Pattnaik , S. Gong , J. Controlled Release 2020, 324, 194. 10.1016/j.jconrel.2020.04.052 PMC7725380 32380204

[82] a) B. Mahaling , D. A. Srinivasarao , G. Raghu , R. K. Kasam , G. Bhanuprakash Reddy , D. S. Katti , Nanoscale 2018, 10, 16485; 29897081 10.1039/c8nr00058a

[83] A. T. Ogunjimi , S. M. G. Melo , C. G. Vargas‐Rechia , F. S. Emery , R. F. V. Lopez , Carbohydr. Polym. 2017, 157, 1065. 27987808 10.1016/j.carbpol.2016.10.076

[84] a) A. Bochot , E. Fattal , J. Controlled Release 2012, 161, 628; 10.1016/j.jconrel.2012.01.019 22289436

[85] L. Li , Z. Zeng , Z. Chen , R. Gao , L. Pan , J. Deng , X. Ye , J. Zhang , S. Zhang , C. Mei , J. Yu , Y. Feng , Q. Wang , A. Y. Yu , M. Yang , J. Huang , ACS Nano 2020, 14, 15403. 33174744 10.1021/acsnano.0c06000

[86] T. Li , Y. Wang , J. Chen , X. Gao , S. Pan , Y. Su , X. Zhou , Drug Delivery 2020, 27, 410. 32133894 10.1080/10717544.2020.1731861 PMC7067192

[87] M. Islam , Y. Xu , W. Tao , J. Ubellacker , M. Lim , D. Aum , G. Lee , K. Zhou , H. Zope , M. Yu , W. Cao , J. Oswald , M. Dinarvand , M. Mahmoudi , R. Langer , P. Kantoff , O. Farokhzad , B. Zetter , J. Shi , Nat. Biomed. Eng. 2018, 2, 850. 31015614 10.1038/s41551-018-0284-0 PMC6486184

[88] Z. Zhou , X. Liu , D. Zhu , Y. Wang , Z. Zhang , X. Zhou , N. Qiu , X. Chen , Y. Shen , Adv. Drug Delivery Rev. 2017, 115, 115. 10.1016/j.addr.2017.07.021 28778715

[89] S. Kim , S. A. Ko , C. G. Park , S. H. Lee , B. K. Huh , Y. H. Park , Y. K. Kim , A. Ha , K. H. Park , Y. B. Choy , Mol. Pharmaceutics 2018, 15, 3143. 10.1021/acs.molpharmaceut.8b00215 30020792

[90] D. Hu , H. Li , B. Wang , Z. Ye , W. Lei , F. Jia , Q. Jin , K. F. Ren , J. Ji , ACS Nano 2017, 11, 9330. 28806528 10.1021/acsnano.7b04731

[91] K. Pollinger , R. Hennig , A. Ohlmann , R. Fuchshofer , R. Wenzel , M. Breunig , J. Tessmar , E. R. Tamm , A. Goepferich , Proc. Natl. Acad. Sci. USA 2013, 110, 6115. 23530216 10.1073/pnas.1220281110 PMC3625317

[92] C. Hu , J. Sun , Y. Zhang , J. Chen , Y. Lei , X. Sun , Y. Deng , Adv. Healthcare Mater. 2018, 7, 1801047. 10.1002/adhm.201801047 30387326

[93] A. V. Cideciyan , S. G. Jacobson , A. V. Drack , A. C. Ho , J. Charng , A. V. Garafalo , A. J. Roman , A. Sumaroka , I. C. Han , M. D. Hochstedler , W. L. Pfeifer , E. H. Sohn , M. Taiel , M. R. Schwartz , P. Biasutto , W. Wit , M. E. Cheetham , P. Adamson , D. M. Rodman , G. Platenburg , M. D. Tome , I. Balikova , F. Nerinckx , J. Zaeytijd , C. Van Cauwenbergh , B. P. Leroy , S. R. Russell , Nat. Med. 2019, 25, 225. 30559420 10.1038/s41591-018-0295-0

[94] G. Chen , A. Abdeen , Y. Wang , P. Shahi , S. Robertson , R. Xie , M. Suzuki , B. Pattnaik , K. Saha , S. Gong , Nat. Nanotechnol. 2019, 14, 974. 31501532 10.1038/s41565-019-0539-2 PMC6778035

[95] K. Kamoi , N. Horiguchi , H. Kurozumi‐Karube , I. Hamaguchi , Y. Yamano , K. Uchimaru , A. Tojo , T. Watanabe , K. Ohno‐Matsui , Lancet Infect. Dis. 2021, 21, 578. 33773136 10.1016/S1473-3099(21)00063-3

[96] B. Burkholder , D. Jabs , BMJ 2021, 372, m4979. 33536186 10.1136/bmj.m4979

[97] L. Luo , J. Yang , Y. Oh , M. J. Hartsock , S. Xia , Y. C. Kim , Z. Ding , T. Meng , C. G. Eberhart , L. M. Ensign , J. E. Thorne , W. J. Stark , E. J. Duh , Q. Xu , J. Hanes , J. Controlled Release 2019, 296, 68. 10.1016/j.jconrel.2019.01.018 PMC6476551 30660629

[98] R. Ganugula , M. Arora , M. A. Lepiz , Y. Niu , M. N. V. R. Kumar , Sci. Adv. 2020, 6, eabb7878. 32923645 10.1126/sciadv.abb7878 PMC7449680

[99] a) Y. Zhou , L. Li , S. Li , S. Li , M. Zhao , Q. Zhou , X. Gong , J. Yang , J. Chang , Nanoscale 2019, 11, 13126; 31268450 10.1039/c9nr02350j

[100] L. Li , J. Guo , Y. Wang , X. Xiong , H. Tao , J. Li , Y. Jia , H. Hu , J. Zhang , Adv. Sci. 2018, 5, 1800781. 10.1002/advs.201800781 PMC6193162 30356945

[101] M. K. Gupta , J. R. Martin , T. A. Werfel , T. Shen , J. M. Page , C. L. Duvall , J. Am. Chem. Soc. 2014, 136, 14896. 25254509 10.1021/ja507626y

[102] T. Kim , K. Sall , E. J. Holland , R. K. Brazzell , S. Coultas , P. K. Gupta , Clin. Ophthalmol. 2019, 13, 69. 30643381 10.2147/OPTH.S185800 PMC6311334

[103] J. Stein , A. Khawaja , J. Weizer , JAMA, J. Am. Med. Assoc. 2021, 325, 164. 10.1001/jama.2020.21899 33433580

[104] a) A. Dillinger , M. Guter , F. Froemel , G. Weber , K. Perkumas , W. Stamer , A. Ohlmann , R. Fuchshofer , M. Breunig , Small 2018, 14, 1803239; 10.1002/smll.201803239 PMC6599181 30353713

[105] T. Stack , M. Vincent , A. Vahabikashi , G. Li , K. M. Perkumas , W. D. Stamer , M. Johnson , E. Scott , Small 2020, 16, 2004205. 10.1002/smll.202004205 PMC7647937 33015961

[106] G. Li , C. Lee , V. Agrahari , K. Wang , I. Navarro , J. Sherwood , K. Crews , S. Farsiu , P. Gonzalez , C. Lin , A. Mitra , C. Ethier , W. Stamer , Proc. Natl. Acad. Sci. USA 2019, 116, 1714. 30651311 10.1073/pnas.1814889116 PMC6358695

[107] D. A. Dik , J. F. Fisher , S. Mobashery , Chem. Rev. 2018, 118, 5952. 29847102 10.1021/acs.chemrev.8b00277 PMC6855303

[108] Y. Qiao , J. He , W. Chen , Y. Yu , W. Li , Z. Du , T. Xie , Y. Ye , S. Y. Hua , D. Zhong , K. Yao , M. Zhou , ACS Nano 2020. 10.1021/acsnano.9b08930 32048825

[109] B. Wang , Z. Ye , Y. Tang , Y. Han , Q. Lin , H. Liu , H. Chen , K. Nan , Int. J. Nanomed. 2017, 12, 111. 10.2147/IJN.S107472 PMC5191580 28053527

[110] B. Duncan , X. Li , R. F. Landis , S. T. Kim , A. Gupta , L. S. Wang , R. Ramanathan , R. Tang , J. A. Boerth , V. M. Rotello , ACS Nano 2015, 9, 7775. 26083534 10.1021/acsnano.5b01696 PMC5047390

[111] C. M. Courtney , S. M. Goodman , J. A. McDaniel , N. E. Madinger , A. Chatterjee , P. Nagpal , Nat. Mater. 2016, 15, 529. 26779882 10.1038/nmat4542

[112] H. Chen , J. Yang , L. Sun , H. Zhang , Y. Guo , J. Qu , W. Jiang , W. Chen , J. Ji , Y. W. Yang , B. Wang , Small 2019, 15, 1903880. 10.1002/smll.201903880 31588682

[113] S. Ling , S. Yang , X. Hu , D. Yin , Y. Dai , X. Qian , D. Wang , X. Pan , J. Hong , X. Sun , H. Yang , S. R. Paludan , Y. Cai , Nat. Biomed. Eng. 2021. 10.1038/s41551-020-00656-y 33398131

[114] a) N. K. Ryoo , J. Lee , H. Lee , H. K. Hong , H. Kim , J. B. Lee , S. J. Woo , K. H. Park , H. Kim , Nanoscale 2017, 9, 15461; 28976519 10.1039/c7nr03142d

[115] S. D. Schwartz , C. D. Regillo , B. L. Lam , D. Eliott , P. J. Rosenfeld , N. Z. Gregori , J. P. Hubschman , J. L. Davis , G. Heilwell , M. Spirn , J. Maguire , R. Gay , J. Bateman , R. M. Ostrick , D. Morris , M. Vincent , E. Anglade , L. V. Del Priore , R. Lanza , Lancet 2015, 385, 509. 25458728 10.1016/S0140-6736(14)61376-3

[116] S. Dehghani , M. Rasoulianboroujeni , H. Ghasemi , S. Keshel , Z. Nozarian , M. Hashemian , M. Zarei‐Ghanavati , G. Latifi , R. Ghaffari , Z. Cui , H. Ye , L. Tayebi , Biomaterials 2018, 174, 95. 29793112 10.1016/j.biomaterials.2018.05.013

[117] a) M. Rizwan , G. S. L. Peh , H. P. Ang , N. C. Lwin , K. Adnan , J. S. Mehta , W. S. Tan , E. K. F. Yim , Biomaterials 2017, 120, 139; 28061402 10.1016/j.biomaterials.2016.12.026

[118] N. Liu , X. Zhang , N. Li , M. Zhou , T. Zhang , S. Li , X. Cai , P. Ji , Y. Lin , Small 2019, 15, 1901907. 10.1002/smll.201901907 31192537

[119] T. Fujie , Y. Mori , S. Ito , M. Nishizawa , H. Bae , N. Nagai , H. Onami , T. Abe , A. Khademhosseini , H. Kaji , Adv. Mater. 2014, 26, 1699. 24307219 10.1002/adma.201304183

[120] a) L. Gu , S. Poddar , Y. Lin , Z. Long , D. Zhang , Q. Zhang , L. Shu , X. Qiu , M. Kam , A. Javey , Z. Fan , Nature 2020, 581, 278; 32433619 10.1038/s41586-020-2285-x

[121] a) L. Everett , T. Copperman , N. Engl. J. Med. 2019, 380, 1853; 31067375 10.1056/NEJMicm1810596

[122] a) R. Gao , R. N. Mitra , M. Zheng , K. Wang , J. C. Dahringer , Z. Han , Adv. Funct. Mater. 2018, 28, 1806248; 32699541 10.1002/adfm.201806248 PMC7375362

[123] K. Jiang , Y. Hu , X. Gao , C. Zhan , Y. Zhang , S. Yao , C. Xie , G. Wei , W. Lu , Nano Lett. 2019, 19, 6410. 31442373 10.1021/acs.nanolett.9b02596

[124] R. Khademi , A. Razminia , Nanomedicine 2020, *24*, 102102. 31678179 10.1016/j.nano.2019.102102

[125] P. G. Hysi , H. Choquet , A. P. Khawaja , R. Wojciechowski , M. S. Tedja , J. Yin , M. J. Simcoe , K. Patasova , O. A. Mahroo , K. K. Thai , P. M. Cumberland , R. B. Melles , V. J. M. Verhoeven , V. Vitart , A. Segre , R. A. Stone , N. Wareham , A. W. Hewitt , D. A. Mackey , C. C. W. Klaver , S. MacGregor , P. T. Khaw , P. J. Foster , J. A. Guggenheim , J. S. Rahi , E. Jorgenson , C. J. Hammond , Nat. Genet. 2020, 52, 401. 32231278 10.1038/s41588-020-0599-0 PMC7145443

[126] T. I. Kim , J. L. A. D. Barrio , M. Wilkins , B. Cochener , M. Ang , Lancet 2019, 393, 2085. 31106754 10.1016/S0140-6736(18)33209-4

[127] S. J. J. Kwok , I. A. Kuznetsov , M. Kim , M. Choi , G. Scarcelli , S. H. Yun , Optica 2016, 3, 469. 28983498 10.1364/OPTICA.3.000469 PMC5626012

[128] L. M. Shaker , A. A. Al‐Amiery , A. A. H. Kadhum , M. S. Takriff , Nanomaterials 2020, 10, 2028. 10.3390/nano10102028 PMC7602513 33076278

[129] C. Wang , M. Fomovsky , G. Miao , M. Zyablitskaya , S. Vukelic , Nat. Photonics. 2018, 12, 416.

[130] M. Wang , Q. Yang , M. Li , H. Zou , Z. Wang , H. Ran , Y. Zheng , J. Jian , Y. Zhou , Y. Luo , Y. Ran , S. Jiang , X. Zhou , ACS Appl. Mater. Interfaces 2020, 12, 5642. 31940169 10.1021/acsami.9b22072

[131] S. M. Evans , K. Kim , C. E. Moore , M. I. Uddin , M. E. Capozzi , J. R. Craft , G. A. Sulikowski , A. Jayagopa , Bioconjugate Chem. 2014, 25, 2030. 10.1021/bc500400z PMC4240343 25250692

[132] V. P. Nguyen , W. Qian , Y. Li , B. Liu , M. Aaberg , J. Henry , W. Zhang , X. Wang , Y. M. Paulus , Nat. Commun. 2021, 12, 34. 33397947 10.1038/s41467-020-20276-z PMC7782787

[133] a) J. Kim , M. Kim , M. Lee , K. Kim , Sa. Ji , Y. T. Kim , J. Park , K. Na , K. H. Bae , H. K. Kim , F. Bien , C. Y. Lee , J. U. Park , Nat. Commun. 2017, 8, 14997; 28447604 10.1038/ncomms14997 PMC5414034

[134] J. Lee , E. J. Jeong , Y. K. Lee , K. Kim , I. C. Kwon , K. Y. Lee , Small 2016, 12, 1201. 26573885 10.1002/smll.201501913

[135] M. Tang , X. Ji , H. Xu , L. Zhang , A. Jiang , B. Song , Y. Su , Y. He , Anal. Chem. 2018, 90, 8188. 29874038 10.1021/acs.analchem.8b01580

[136] Y. Lin , Y. Zhang , J. Li , H. Kong , Q. Yan , J. Zhang , W. Li , N. Ren , Y. Cui , T. Zhang , X. Cai , Q. Li , A. Li , J. Shi , L. Wang , Y. Zhu , C. Fan , Nano Today 2020, 35, 100922.

[137] X. Chen , F. Wang , J. Y. Hyun , T. Wei , J. Qiang , X. Ren , I. Shin , J. Yoon , Chem. Soc. Rev. 2016, 45, 2976. 27092436 10.1039/c6cs00192k

[138] Z. Wu , J. Troll , H. H. Jeong , Q. Wei , M. Stang , F. Ziemssen , Z. Wang , M. Dong , S. Schnichels , T. Qiu , P. Fischer , Sci. Adv. 2018, 4, eaat4388. 30406201 10.1126/sciadv.aat4388 PMC6214640

[139] Z. Tang , F. Jiang , Y. Zhang , Y. Zhang , YuanYang, X. H. , Y. Wang , D. Zhang , N. Ni , F. Liu , M. Luo , X. Fan , W. Zhang , P. Gu , Biomaterials 2019, 194, 57. 30583149 10.1016/j.biomaterials.2018.12.015

[140] Y. C. Kim , M. D. Shin , S. F. Hackett , H. T. Hsueh , E. S. R. Lima , A. Date , H. Han , B. J. Kim , A. Xiao , Y. Kim , L. Ogunnaike , N. M. Anders , A. Hemingway , P. He , A. S. Jun , P. J. McDonnell , C. Eberhart , I. Pitha , D. J. Zack , P. A. Campochiaro , J. Hanes , L. M. Ensign , Nat. Biomed. Eng. 2020, 4, 1053. 32895514 10.1038/s41551-020-00606-8 PMC7655548

[141] D. Kim , H. Lee , S. Kwon , Y. Sung , W. Song , S. Park , Adv. Healthcare Mater. 2020, 9, 2000118. 10.1002/adhm.202000118 32431072

